# Integrating Multi-Omics Summary Data Identifies Candidate Molecular Mechanisms for Major Depression

**DOI:** 10.1016/j.biopsych.2025.11.024

**Published:** 2025-12-15

**Authors:** Laurence Nisbet, Yang Wu, Mark Adams, Mary-Ellen Lynall, Jens Hjerling-Leffler, Naomi R. Wray, Andrew M. McIntosh, Xueyi Shen

**Affiliations:** Division of Psychiatry, University of Edinburgh, Edinburgh, United Kingdom; Institute of Rare Diseases, West China Hospital of Sichuan University, Chengdu, China; Division of Psychiatry, University of Edinburgh, Edinburgh, United Kingdom; Department of Psychiatry, University of Oxford, Oxford, United Kingdom; Department of Psychiatry, University of Cambridge, Cambridge, United Kingdom; Department of Medical Biochemistry and Biophysics, Karolinska Institute, Stockholm, Sweden; Department of Psychiatry, University of Oxford, Oxford, United Kingdom; Institute for Molecular Bioscience, University of Queensland, Brisbane, Queensland, Australia; Division of Psychiatry, University of Edinburgh, Edinburgh, United Kingdom; Division of Psychiatry, University of Edinburgh, Edinburgh, United Kingdom

## Abstract

**BACKGROUND:**

Major depression (MD) is the most common psychiatric disorder. However, despite having a significant genetic component, the underlying biological mechanisms remain poorly understood. Our analyses leveraged molecular quantitative trait loci (xQTL) data to identify molecular biomarkers for MD.

**METHODS:**

We used OPERA (Omics Pleiotropic Association) software to identify molecular phenotypes associated with MD through shared causal variants, using genome-wide association study (GWAS) summary statistics and xQTL data for 5 phenotypes in blood and brain tissues. The xQTL phenotypes were gene expression, DNA methylation, splicing variation, chromatin accessibility, and protein abundance.

**RESULTS:**

We identified 939 genes in blood tissues and 607 genes in brain tissues associated with MD via at least 1 molecular phenotype. Drug targets were enriched in our significant genes in both tissues. A total of 23 genes showed associations via 3 or more molecular phenotypes, providing robust evidence for their causal role in MD and offering insights into their biomolecular mechanisms. These high-priority associations included genes that have been previously identified by GWASs of MD such as *CDH13* and *RAB27B* as well as novel associations such as *H6PD*.

**CONCLUSIONS:**

Our results highlight promising new targets for biomarker and drug target identification and successfully expand on GWAS findings to identify novel associations with MD. However, our study took a broad approach using bulk brain and blood tissues. Future research should expand these analyses into cell- and region-specific contexts.

Depression is the most common psychiatric disorder and a leading cause of disability worldwide (1,2). For many patients, the condition is partially resistant to antidepressants and other treatments (3). There is a notable genetic risk component, and genome-wide association studies (GWASs) have had considerable success identifying genetic variants significantly associated with major depression (MD) (4–6). However, the biomolecular mechanisms through which these variants affect MD remain poorly understood. Improving the translation of genetic findings into molecular mechanisms and clinical interventions has therefore become a major priority.

Functional genomics approaches, such as studying biomolecular phenotypes to understand genomic mechanisms, have become a promising area of research seeking to understand the role of genetics in disease (7–9). Examples of these molecular phenotypes (also called omics types) include proteomics (proteins), transcriptomics (gene expression), and epigenomics (e.g., DNA methylation). Understanding the role these molecular phenotypes play in MD risk is essential in determining how GWAS hits correspond to biological mechanisms and potentially to clinically relevant biomarkers and drug targets.

A common approach to link risk variants to biological mechanisms uses molecular quantitative trait loci (xQTL) analysis, which connects the association of genetic variants to the expression or abundance of specific molecular phenotypes. Examples include xQTL for protein abundance and for gene expression. xQTL data can be integrated with GWASs in downstream analyses such as Mendelian randomization (MR) (10,11). MR uses genetic instruments to estimate the effect one phenotype (the exposure) has on another (the outcome) by testing whether the exposure and the outcome are influenced by the same causal genetic variants. Treating molecular phenotypes such as gene expression as the exposure and a psychiatric disorder as the outcome allows researchers to identify the molecular mechanisms underlying GWAS relationships with psychiatric disorders (12).

MR studies have implicated a range of molecular phenotypes in MD, including gene expression (10,13), protein abundance (14,15), and DNA methylation (16,17), and have used these molecular markers to implicate specific genes. However, these studies have limitations; the vast majority examine only 1 or 2 molecular phenotypes at a time and attempt to integrate these in a hierarchical or stepwise manner. This increases the number of tests, leading to a loss of power and a greater multiple testing burden relative to an integrative method, which incorporates multiple molecular phenotypes into a single analysis (7,18). Additionally, this approach provides only a limited snapshot of the layered molecular interactions through which genetic variants can affect a trait. Furthermore, in contrast to genetic variation, the abundance and function of molecular phenotypes may differ between tissues, and so xQTL can be tissue dependent (19). The majority of previous studies have used blood, with a smaller number using more relevant tissues of postmortem brain or cerebrospinal fluid (20–22). A small number have conducted analyses across both blood and brain tissues, with one study using xQTL identifying one gene (*TMEM106B*) significantly associated with MD across blood and brain tissues (14).

Our study aimed to address these limitations using OPERA (Omics Pleiotropic Association) software (18) by leveraging multiple recently published molecular xQTL datasets in both brain and blood tissues, which offer increased power compared with previous studies. OPERA builds on MR by using shared genetic variants as instruments to integrate multiple molecular phenotypes (as exposures) into a single analysis. It outputs groupings of molecular markers (configurations) that, collectively, are significantly associated with a complex trait. When significant molecular markers all map to one gene, we are able to identify likely instances of vertical pleiotropy, in which genetic variants affect complex traits via their effects on a cascade of multiple molecular phenotypes (probes) within a locus (e.g., individual protein abundance or measures of DNA methylation).

We applied OPERA to test for associations with MD across 5 different molecular phenotypes, i.e., using xQTL for protein abundance, gene expression, splicing variation, DNA methylation, and chromatin accessibility in both the blood and the brain, to identify multi-omics biomarkers for MD. We additionally tested whether drug targets were enriched in our results. Finally, we conducted OPERA analyses using schizophrenia and bipolar disorder (BPD) as the outcome traits and compared the results with the MD analysis to identify potential causal genes shared between the 3 disorders. Our analysis identified candidate genes associated with MD alongside multiple molecular phenotypes that were potentially mediating the gene-trait associations.

## METHODS AND MATERIALS

### GWAS Summary Statistics for Psychiatric Disorders

We leveraged summary statistics from GWASs of MD (*n* = 685,808 cases, *n* = 4,364,225 controls, number of independent genome-wide significant [iGWS] single nucleotide polymorphisms [SNPs] = 697) (6), schizophrenia (*n* = 76,755 cases, *n* = 243,649 controls, number of iGWS SNPs = 287) (23), and BPD (*n* = 41,917 cases, *n* = 371,549 controls, number of iGWS SNPs = 64) (24). For the blood-based analyses, we used summary statistics calculated excluding the UK Biobank (UKB) sample to avoid overlap with the xQTL for protein abundance blood sample, which was also derived from the UKB: for MD, *n* = 401,750 cases, *n* = 2,822,566 controls (6); for schizophrenia, *n* = 67,323 cases, *n* = 93,456 controls (23); and for BPD, *n* = 40,463 cases, *n* = 313,436 controls (24).

All three datasets were obtained from the Psychiatric Genomics Consortium (PGC) (https://pgc.unc.edu). In the MD GWAS, the phenotype was defined using a combination of electronic health records, clinical interviews, and self-reported symptoms (for details, see Supplemental Methods). Full details on processing, quality control, and cohort profiles are provided in the respective studies.

We filtered SNPs to remove duplicates, multiallelic SNPs, and those with a minor allele frequency <0.05 or an INFO score <0.90. The X chromosome was excluded from analysis. The BPD GWAS consisted of cohorts of European ancestry only. The MD and schizophrenia GWASs were conducted in predominantly European cohorts but included several cohorts from other ancestries [see (6) and (23) for details]. We used the European sample of the 1000 Genomes Project as the linkage disequilibrium (LD) reference panel (*n* = 503) (25).

### xQTL Summary Statistics for Molecular Phenotypes

We collected the largest available xQTL summary statistics for 5 molecular phenotypes in both blood and brain tissues (Table 1). We refer to individual measures of a molecular phenotype, for example, specific proteins or gene expression transcripts, as probes. In total, we collected data detailing the association between molecular phenotypes and *cis*-genetic variants for 822,235 probes in the brain and 485,619 probes in the blood. All xQTL datasets were collected from predominantly European samples. The datasets varied in coverage between approximately 3 million and 8.5 million SNPs. All genome builds were aligned to GRCh37/hg19.

The xQTL datasets are available from the Functional Genomics group of the PGC (https://pgc.unc.edu/for-researchers/working-groups/functional-genomics-working-group/). For further information on the xQTL data, see Supplemental Methods.

### Multi-omics Association Analysis

Our primary analysis used OPERA software developed by Wu *et al.* (18). OPERA uses GWAS and xQTL data to identify potentially causal effects of multiple molecular phenotypes (exposures) on MD (the outcome) with the aim of identifying biomolecular mechanisms for GWAS signals.

The software outputs configurations of significant omics probes, which share causal SNPs and the *cis*-regulated variation of which is collectively associated with MD. Configurations are limited to 1 probe per molecular phenotype; therefore the largest possible configuration in our analysis could contain 5 omics probes. OPERA also uses an LD reference to run the heterogeneity in dependent instruments (HEIDI) test to distinguish the pleiotropic associations from linkage (26). Configurations with a posterior probability of association (PPA) > 0.90, and a *p*_HEIDI_ > .01 were considered to be significant (18). OPERA limits analysis to SNPs within 1 Mb of independent GWS SNPs (*p* < 5 × 10^−8^; MD = 697, schizophrenia = 287, BPD = 64) due to the high computational burden of a full Bayesian analysis and to focus on regions with a greater likelihood of functional associations (18). For a full explanation of the function and interpretation of OPERA, see Supplemental Methods and refer to Wu *et al.* (18).

In our primary analysis, we tested for causal relationships between molecular phenotypes and MD. Analyses of schizophrenia (23) and BPD (24) were added as supplemental analyses. We applied OPERA separately to blood and brain xQTL datasets. Across all 10 xQTL datasets, the 485,619 omics probes from blood-derived xQTL datasets mapped to 36,842 genes, and the 822,235 omics probes from brain-derived xQTL datasets mapped to 35,682 genes. We mapped significant omics probes to genes to identify those contributing to MD risk (see Supplemental Methods).

### Identifying Druggable Genes

We tested whether the druggable genome was enriched in our significant genes. The druggable genome was defined as the 4160 genes listed as drug targets in the DrugBank version 5.1.12 database (27). We downloaded similar lists for genes listed as carriers, enzymes, and transporters. We conducted 1-tailed hypergeometric tests on our lists of significant genes in the brain and blood to test whether overlaps between DrugBank-identified genes and our significant gene lists were greater than expected. We conducted tests separately against the DrugBank list of genes for targets, carriers, enzymes, and transporters (Table S1). We used background gene lists, one each for blood and brain tissues, which included all mapped genes tested in our analysis for each tissue.

### Comparing Molecular Phenotypes Between Tissues

For each molecular phenotype, we calculated the proportion of probes that were significant in our analysis. We plotted these to assess whether the proportion of significant probes in each molecular phenotype differed between brain and blood tissues (Figure S1).

Using Fisher’s exact test, we determined whether there was a significant difference in the distribution of configuration size between blood and brain tissues. The input was a 2 × 4 contingency table containing frequencies of configuration sizes for brain and blood tissues (Table S2).

### Overlap in Significant Genes Between MD, BPD, and Schizophrenia

We hypothesized that there would be significant overlap in genes identified from the OPERA analyses of MD, schizophrenia, and BPD. We used pairwise hypergeometric tests to determine whether the overlaps were significantly greater than chance (Table S3). As with the drug target analysis, background gene lists consisted of the genes mapped from all probes included in the analysis. Lastly, we calculated the Pearson correlation between the summary data–based Mendelian randomization (SMR) effect sizes of the 3 disorders (SMR effect sizes were calculated using OPERA as part of its first step).

## RESULTS

### Genes With Multi-Omics Associations With MD

**Figure 1.**
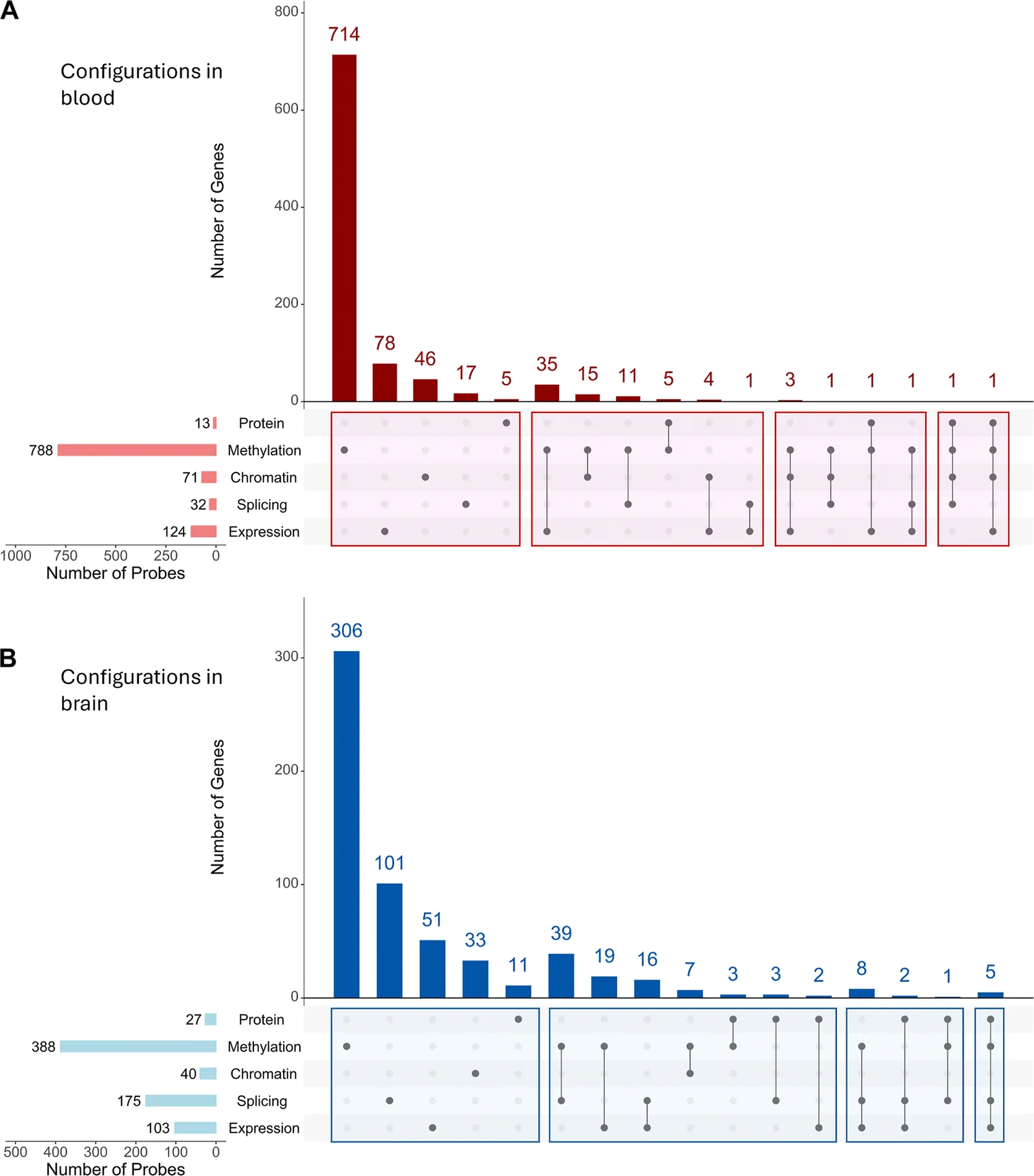
UpSet plots depicting the number of genes for which different configuration types were found in **(A)** blood and **(B)** brain tissues. This figure represents only the different types of configurations by probe type, rather than the number of significant configurations (57,58)

Using blood-based xQTL, we identified 939 genes that showed evidence of contributing to MD risk via *cis*-regulated variation in at least 1 omics type (PPA > 0.90 and *p*_HEIDI_ > .01). Of 939 genes, 2 showed associations across 4 omics types, 6 showed associations across 3 types, 71 showed associations across 2 types, and 860 showed associations across 1 type (Figure 1A). These were mapped from 1877 probes that showed an association with MD in 4953 significant configurations.

Using brain-based xQTL, we identified 607 genes that showed evidence of contributing to MD risk via *cis*-regulated variation in at least 1 omics type (PPA > 0.90 and *p*_HEIDI_ > .01). Of 607 genes, 5 showed associations across 4 omics types, 11 showed associations across 3 types, 89 showed associations across 2 types, and 502 showed associations across 1 type (Figure 1B). These were mapped from 1405 probes that showed an association with MD in 3737 significant configurations.

There were 334 genes significantly associated with MD in both brain and blood tissues. We found that the distribution of configuration sizes differed significantly between brain and blood tissues (2-tailed Fisher’s exact test, *p* < 2.2 × 10^−16^). While a greater number of configurations were significant in blood tissues, configurations were on average larger (i.e., implicated in more molecular phenotypes) in brain tissues.

**Figure 2.**
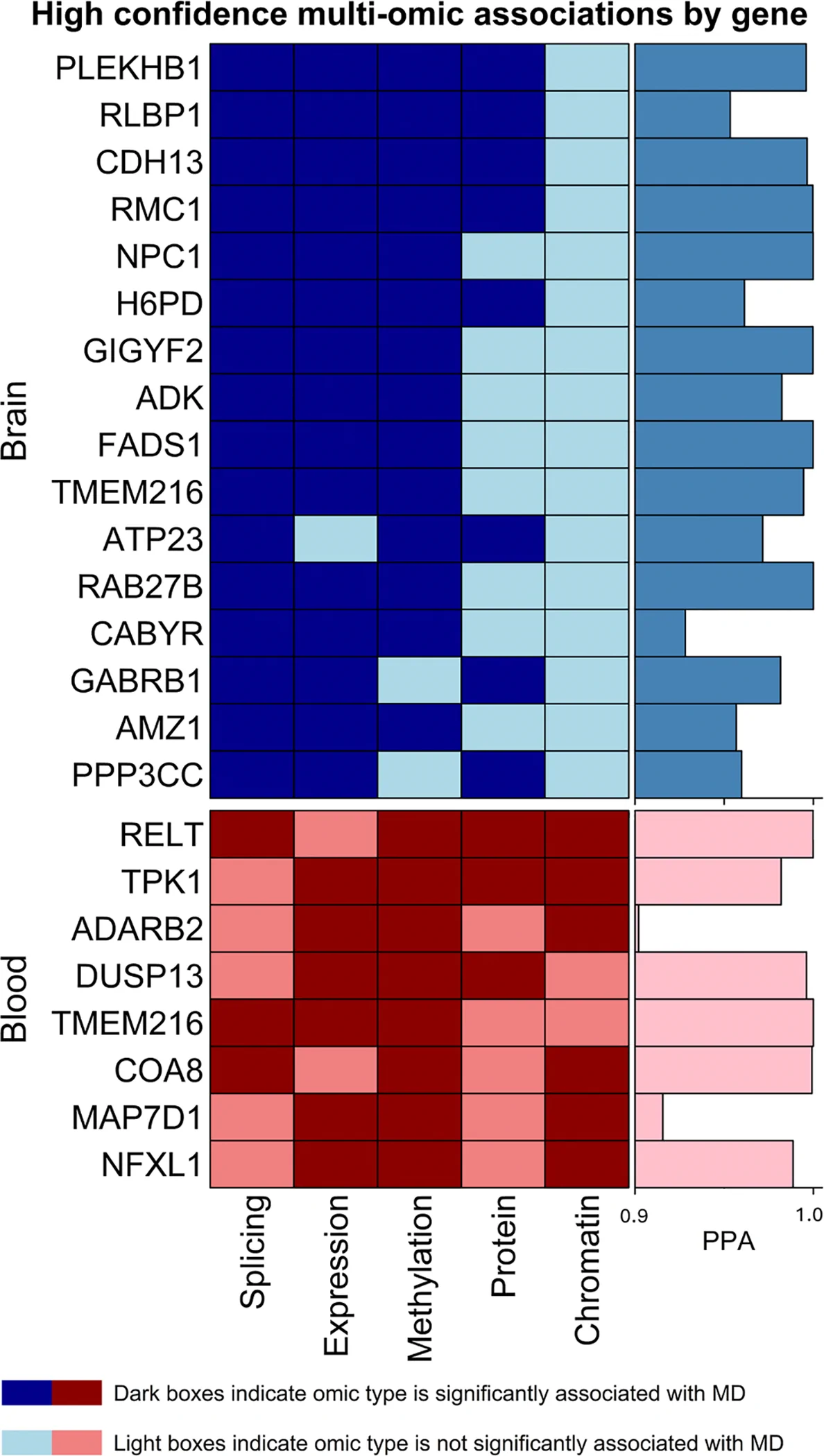
Two-tone heatmap summarizing patterns of significant association in high-confidence genes. Genes are shown on the y-axis, and omic phenotypes are shown on the x-axis. Dark boxes indicate that for a given gene, at least 1 omic marker of that type is significantly associated with major depression (MD). High-confidence genes were defined as those for which we observed causal associations with MD at 3 or more omic layers. Significant configurations of associated omic markers were defined as those with a posterior probability of association (PPA) > 0.90.

**Figure 3.**
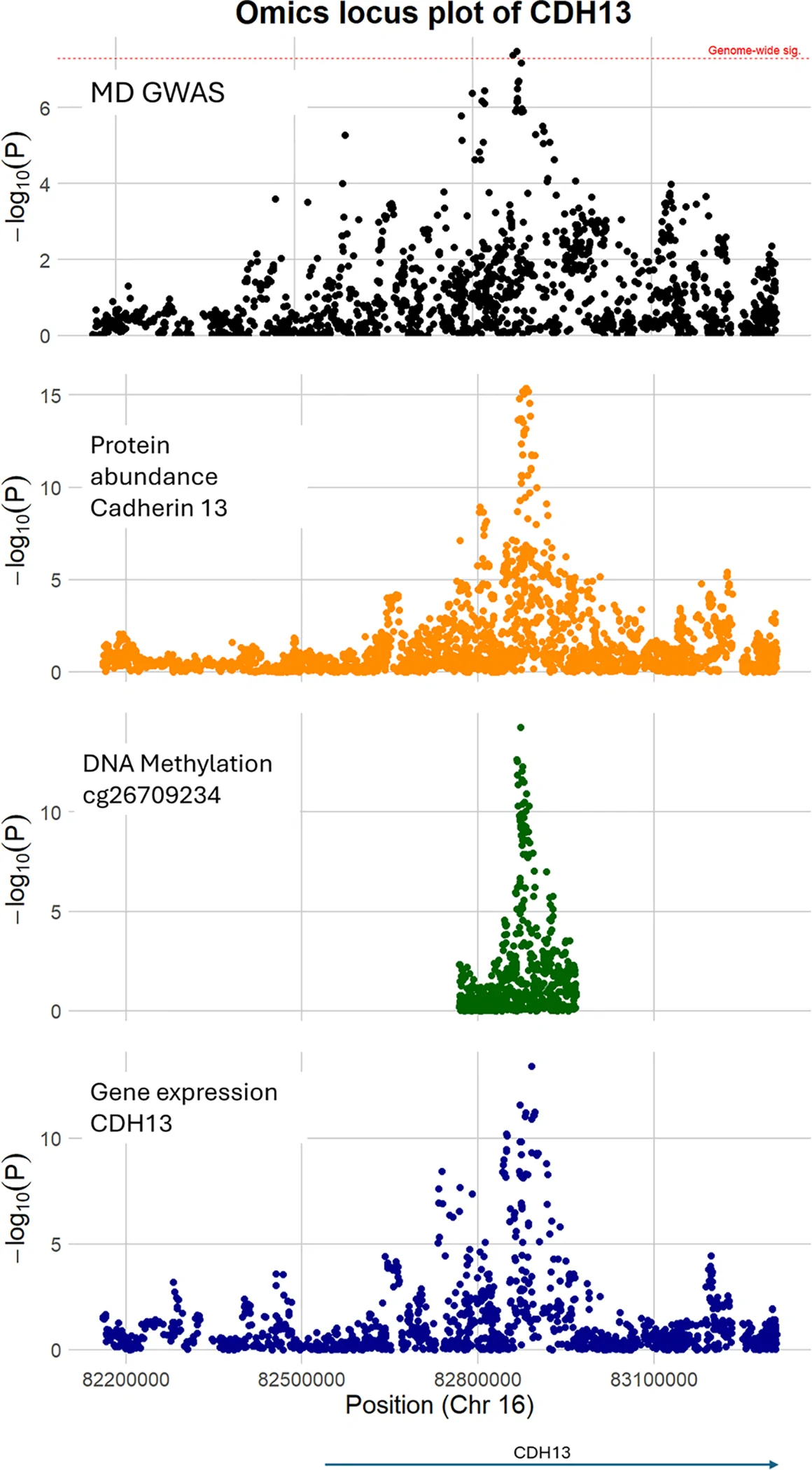
Example multi-omic summary data–based Mendelian randomization plot of *CDH13* demonstrating how molecular quantitative trait loci associations in the brain align across multiple omic phenotypes. *CDH13* was selected as an example of a gene showing vertically pleiotropic signals across 4 omic phenotypes, identified using OPERA (Omics Pleiotropic Association) with a posterior probability of association > .90. Chr, chromosome; GWAS, genome-wide association study; MD, major depression; sig., significant.

We classed the 23 genes supported by associations with MD at 3 or more omics layers as high-priority genes. Of these genes, 16 were associated with MD via brain-derived molecular phenotypes (*PLEKHB1, RLBP1, CDH13, RMC1, NPC1, H6PD, GIGYF2, ADK, FADS1, TMEM216, ATP23, RAB27B, CABYR, GABRB1, AMZ1,* and *PPP3CC*), and 8 were associated with MD via blood-derived phenotypes (*RELT, TPK1, ADARB2, DUSP13, TMEM216, COA8, MAP7D1,* and *NFXL1*). Note that *TMEM216* was significant across 3 omics types in both brain and blood (Figure 2). Locus plots (Figure 3; Figure S2) show examples visualizing how the raw data imply vertical pleiotropy for significant genes.

### Identifying Potential Drug Targets

DrugBank listed 2803 of the genes included in our analysis as targets (27). Of the 939 genes significant across at least 1 omics layer in the blood-based OPERA analysis, 105 (11.18%) were listed as targets. In the brain-based analysis, 70 of the 607 significant genes (11.53%) were listed as targets. Following Bonferroni correction (*p* < .013 to be considered significant), hypergeometric tests determined drug targets to be significantly enriched in the gene sets identified from the OPERA analysis (*p* = 4.84 × 10^−5^ in blood tissues and *p* = 8.24 × 10^−4^ in brain tissues).

DrugBank listed 419 of the genes tested in our blood-based analysis and 421 of the genes tested in the brain-based analysis as enzyme-coding genes (27). From the gene set identified from the OPERA analysis in blood tissues, 19 of the 939 significant genes (2.03%) were listed as enzyme-coding genes, and in the brain, 14 of the 607 significant genes (2.31%) were listed as enzyme-coding genes. Following Bonferroni correction (*p* < .013 to be considered significant), hypergeometric tests determined enzyme-coding genes to be significantly enriched in the gene sets identified from the OPERA analysis in blood (*p* = .012), but not in the brain (*p* = .014). A similar analysis of transporters and carriers found that neither were significantly enriched in either blood or brain. For a full breakdown of the hypergeometric test inputs, see Table S1.

Among our 23 high-priority genes, DrugBank classified *TPK1* in blood tissues and *H6PD, FADS1, GABRB1,* and *ADK* in brain tissues as drug targets. *TPK1, FADS1,* and *ADK* were also classified as enzyme-coding genes. Additionally, *RLBP1* in brain tissues was listed as a carrier (27,28). In all genes except *FADS1,* increased gene expression was positively associated with MD, so the findings suggest that drugs inhibiting expression of these genes would have beneficial effects.

### Comparison With Schizophrenia and BPD

In addition to MD, OPERA analyses of BPD and schizophrenia were performed. Ten genes showed significant associations in both tissue types and across all 3 disorders (*ITIH1, NT5DC2, UQCC5, STAB1, STIMATE, STIMATE-MUSTN1, RP5–966M1.6, CACNA1C, NEK4,* and *MAD1L1*).

#### Blood-Based Analysis.

**Figure 4.**
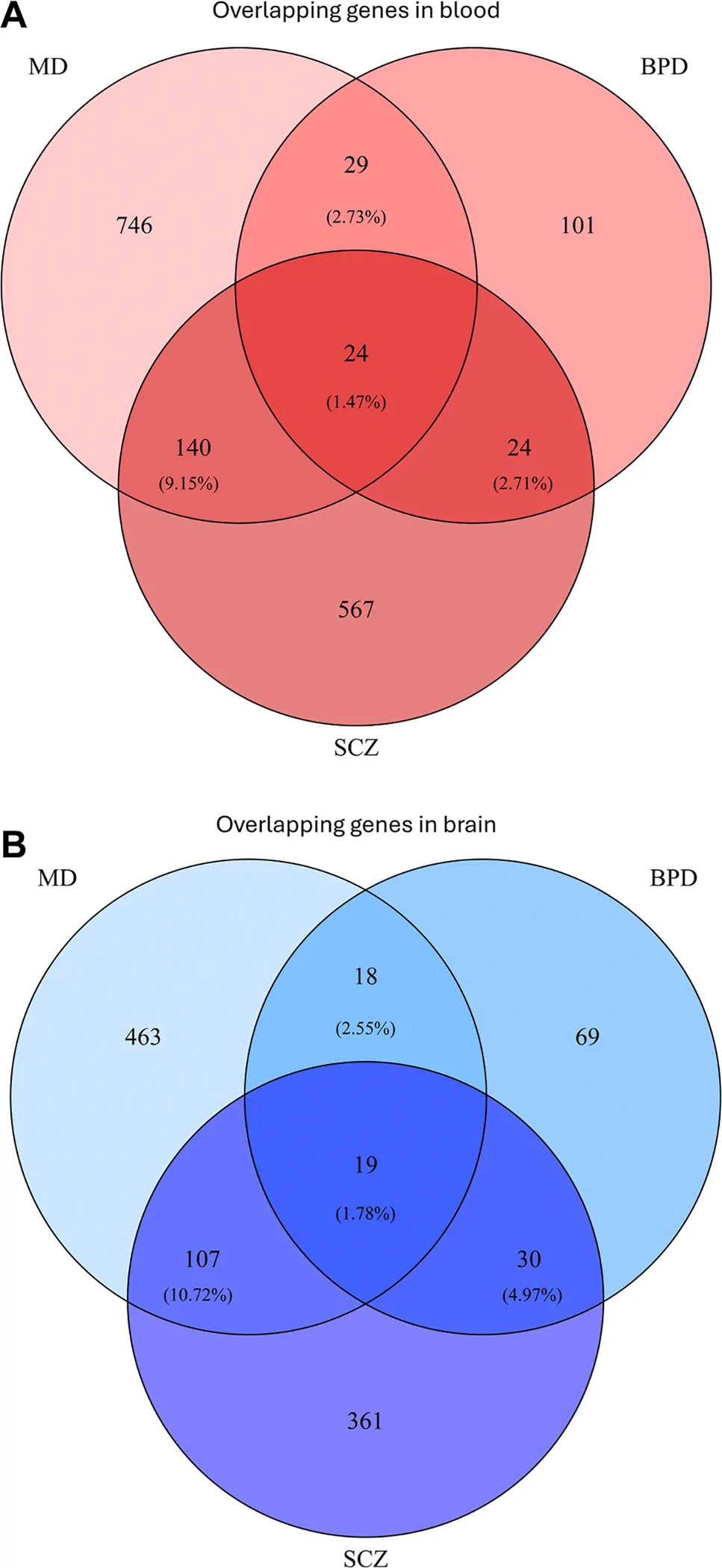
Overlap of genes mapped from significant OPERA (Omics Pleiotropic Association) probes across different psychiatric phenotypes in **(A)** blood and **(B)** brain tissues. Percentages reflect the proportion of the pairwise total of genes shared between gene sets. A full breakdown of which genes were associated with multiple disorders is provided in Table S4. BPD, bipolar disorder; MD, major depression; SCZ, schizophrenia.

For schizophrenia, 1459 probes were significant and mapped to 755 genes across 162 loci. In BPD, 423 probes were significant and mapped to 178 genes across 43 loci. Comparing the mapped genes with those from the MD analysis, 164 genes overlapped between schizophrenia and MD, 53 genes overlapped between BPD and MD, and 48 genes overlapped between schizophrenia and BPD. Lastly, there was an overlap of 24 genes between all 3 disorders (Figure 4A; Table S4).

Hypergeometric tests showed significant overlap between all 3 disorders (*p* < .017 was considered significant): between MD and schizophrenia, *p* = 7.62 × 10^−104^; between MD and BPD, *p* = 3.10 × 10^−41^; and between schizophrenia and BPD, *p* = 1.33 × 10^−39^ (Table S3). The joint SMR results from the blood-based analyses were positively correlated between all 3 disorders: *r*_MDD~SCZ_ = 0.38, *r*_MDD~BPD_ = 0.44, *r*_SCZ~BPD_ = 0.74 (Figures S3–S5).

For schizophrenia, we identified 15 high-confidence genes in the blood, 2 of which, *COA8* and *MAP7D1*, overlapped with the MD high-confidence genes. For BPD, we identified no high-confidence genes in the blood (Figures S9 and S10).

#### Brain-Based Analysis.

For schizophrenia, 1077 probes were significant that mapped to 517 genes across 140 loci. For BPD, 344 probes were significant that mapped to 136 genes across 35 loci. Comparing the mapped genes with those from the MD analysis, there was an overlap of 126 genes between the schizophrenia and MD results, an overlap of 37 genes between the BPD and MD results, and an overlap of 49 genes between schizophrenia and BPD. Lastly, there was an overlap of 19 genes between all 3 disorders (Figure 4B; Table S4).

Hypergeometric tests showed significant overlap between all 3 disorders (*p* < .017 to be considered significant): between MD and schizophrenia, *p* = 1.24 × 10^−108^; between MD and BPD, *p* = 6.90 × 10^−34^; and between schizophrenia and BPD, *p* = 7.09 × 10^−55^ (Table S3). The joint SMR results from the brain-based analyses were positively correlated between all 3 disorders: *r*_MDD~SCZ_ = 0.24, *r*_MDD~BPD_ = 0.42, *r*_SCZ~BPD_ = 0.74 (Figures S6–S8).

For schizophrenia, we identified 14 high-confidence genes in the brain, 1 of which, *RAB27B*, overlapped with the MD high-confidence genes. For BPD, we identified 6 high-confidence genes in the brain, 1 of which, *FADS1*, was shared with MD (Figures S9 and S10).

## DISCUSSION

Our study sought to identify genes that conferred the effects of risk variants for MD on the disorder via multiple molecular phenotypes. Using the OPERA tool, we identified 939 genes using blood-based xQTL that showed a significant association with MD in at least 1 molecular phenotype. Of these genes, 79 showed an association across multiple molecular phenotypes. We identified 607 genes using brain-based xQTL that showed a significant association with MD across 1 or more molecular phenotypes. Of these genes, 105 showed an association across multiple molecular phenotypes. Lastly, 334 genes showed associations in both blood and brain across at least 1 molecular phenotype. Functional annotation analysis of these genes found that they were enriched in sets related to cell adhesion, neuronal development, and synapse assembly in line with previous studies (5,15,29,30).

A total of 23 genes showed a significant association with MD across 3 or more molecular phenotypes, allowing us to trace genetic effects through cascading molecular mechanisms (i.e., vertical pleiotropy) (Figure 2). A number of these genes have previously been implicated at only 1 or 2 omics layers including *RAB27B* and *FADS1* (14–16,30–32). In addition, some novel genes were identified, such as *H6PD*. These genes have a range of functions, including synaptic inhibition (*GABRB1*) and intracellular cholesterol transportation (*NPC1*) (33,34). Of particular note, *TMEM216* was significant across 3 omics types in both blood and brain tissues and is involved in ciliogenesis (35).

Compared with previous studies, our findings represent a significant increase in the number of associations across multiple molecular phenotypes and build on previous findings with richer detail of the multi-omics signatures of known risk genes (9,13). For example, 2 studies integrating proteomic and transcriptomic data identified only 4 and 5 genes, respectively, that were significantly associated with MD and that replicated across both omics types (14,15). Additionally, most previous studies incorporated no more than 2 molecular phenotypes compared with the 5 phenotypes in our study, and studies that included more had a loss of power due to an increased testing burden (12,14,15,36).

The increased power and ability to implicate multiple molecular mechanisms in this study has implications for both biomarker identification and drug development. In particular, the need for novel drug targets for MD has been strongly emphasized (37), given that many patients do not respond to currently available treatments (3,38). Drug targets were significantly enriched in our lists of significant genes from the OPERA analysis for both blood and brain tissues. Among our 23 high-priority genes, 5 were listed as targets in the DrugBank data library (*TPK1, H6PD, FADS1, GABRB1,* and *ADK*). Drug target analysis indicated that none of these genes were yet directly implicated in MD, although closely related products such as GABA_A_ (gamma-aminobutyric acid A) receptors have been shown to play a role (39). Our results demonstrate the use of OPERA in identifying novel pharmacological treatments for MD, and the multi-omics information it produces could be used to identify where existing drugs can be repurposed in treating MD.

Comparing the relative biological importance of each molecular phenotype between tissues in our results is challenging; while there was a greater likelihood of observing multi-omics effects in brain tissues due to the same tissues being used in multiple omics measures, findings observed in blood tissues were less prone to confounding as they were observed across independent samples and had a greater effective sample size. Despite this, we found good concordance between brain- and blood-based results. In particular for gene expression and protein abundance, a similar proportion of probes was significant in brain and blood tissues despite much smaller samples in the brain xQTL datasets. This likely reflects the special relevance of neural tissue for psychiatric disorders, although the higher overlap in donors between brain QTL datasets relative to blood is a contributing factor.

In addition to MD, we conducted OPERA analyses of schizophrenia and BPD using the same molecular phenotype datasets (23,24). We observed molecular phenotype associations across 517 genes in brain tissues and 755 genes in blood tissues for schizophrenia and 136 genes in brain tissues and 178 genes in blood tissues in BPD. Previous studies found significant genetic correlation between these disorders (40,41). Consistent with these, our analyses revealed significant overlap in implicated genes between the 3 traits. Additionally, the correlations between SMR effects of molecular phenotypes on the disorders broadly aligned with the reported genetic correlations (41). Our overlapping genes were implicated in a diverse range of mechanisms, including calcium signaling (*CACNA1C*) and cell division (*MAD1L1*) (42,43). As noted above, there was significant overlap in genes associated in at least 1 omics type across all 3 disorders. However, there was limited overlap between genes significant across 3 or more omics types and multiple disorders; only *RAB27B* was significant across 3 omics types in MD and schizophrenia and *FADS1* was significant between MD and BPD. As expected, considering the genetic correlation between the 3 disorders, our results capture both MD-specific effects and effects relevant to other psychopathologies.

We acknowledge a few limitations to our study. In particular, our approach prioritizes sample size and statistical power over specificity. For example, we did not apply age- or sex-restricted xQTL datasets due to limited availability of summary data. In addition, to improve comparability across xQTL studies, we opted for bulk brain–derived data over brain region– or cell type–specific analysis. To determine the impact of our novel associations on drug and biomarker discovery, future research should investigate their role in specific tissues and cell networks. Cell type–specific analysis, in particular, is an important area. Understanding of the underlying cell-specific mechanisms is needed to understand the functional consequences of risk-associated genetic variation (44).

Further, we note that while the MD GWAS drew on a variety of phenotyping methods, what effect this has on our results, if any, is unclear (6). However, we note that genetic architecture of MD remains highly correlated across different definitions (e.g., *r*_g_ = approximately 0.85 between clinically ascertained and self-reported definitions) (6,45). Additionally, we are limited by the uneven coverage of molecular markers across the genome; for example, only approximately 8000 proteomic probes were available in the brain compared with more than 18,000 gene expression probes. Defining high-confidence genes by significance across any 3 molecular phenotypes accommodates in part for this; however, there may be many relevant multi-omics signals not captured by this analysis. This imbalance may also contribute to the high rate of single omics associations (see Supplemental Methods). Similarly, the available sample sizes for brain-derived xQTL were considerably smaller than those for blood; there was also increased sample overlap between xQTL datasets owing to the scarcity of postmortem brain samples (see Supplemental Methods). Nonetheless, we were still able to detect numerous significant multi-omic associations in the brain, comparable to the blood-based samples. Our study highlights the importance of harmonizing and generating high-quality QTL data across multiple molecular phenotypes, which can potentially accelerate functional annotation to GWAS findings. Finally, the datasets used in this analysis were drawn from European samples. There is likely considerable overlap in functional SNPs across ancestries. Therefore, including more diverse samples is important for the generalizability of our findings and for identifying causal SNPs using techniques such as MESUSie (46).

## Conclusions

Through the OPERA software, we were able to incorporate multiple molecular phenotypes into a single analysis testing for associations between molecular measures and MD. This offers both improved power and improved resolution over single-layer methods. We were able to identify a significant number of genes associated with MD across multiple molecular phenotypes as well as assess their relevance as potential biomarkers and drug targets.

## Supplementary Material

1

2

Supplementary material cited in this article is available online at https://doi.org/10.1016/j.biopsych.2025.11.024.

## Figures and Tables

**Table 1.** xQTL Datasets Used in the Study After Quality Control

Study (Dataset)	Omics Type	Tissue Type	Sample Size	No. of Probes	Age, Years
Sun*et al*., 2023 (47) (UK Biobank)	Protein	Blood plasma	54,219	2831	40–69
Wingo *et al*., 2023 (48) (ROSMAP)	Protein	Bulk brain	1277	8044	61–108
Qi *et al*., 2018 (49) (BrainMeta)^a^	DNA methylation	Bulk brain	1160	432,106	0–108
Min *et al*., 2021 (50) (GoDMC)	DNA methylation	Whole blood	32,851	208,699	0–98
Võsa *et al*., 2021 (51) (eQTLgen 1 Consortium)	Gene expression	Whole blood + PBMCs	31,684	19,226	17–88
de Klein *et al*., 2023 (52) (MetaBrain Consortium)	Gene expression	Brain (cortex)	2683	18,387	16–108
Li *et al*., 2018 (53) (GTEx)	Gene splicing	Whole blood	670	65,109	20–71
Qi *et al*., 2022 (54) (BrainMeta)^b^	Gene splicing	Bulk brain	2443	61,507	0–108
Kumasaka*et al*., 2019 (55)	Chromatin accessibility	Blood (lymphocytes)	100	254,863	≥18
Zeng *et al*., 2024 (56)	Chromatin accessibility	Bulk brain (neurons)	616	302,191	18–108

Note that some xQTL datasets had sample overlap, e.g., the ROSMAP and GTEx samples were used in multiple studies. We used analysis options within OPERA (Omics Pleiotropic Association) that account for correlations between datasets (see Supplemental Methods).

GoDMC, Genetics of DNA Methylation Consortium; GTEx, Genotype-Tissue Expression; PBMC, peripheral blood mononuclear cell; ROSMAP, Religious Orders Study and Memory and Aging Project; xQTL, molecular quantitative trait loci.

a See https://yanglab.westlake.edu.cn/software/smr/#mQTLsummarydata.

b See https://yanglab.westlake.edu.cn/data/brainmeta/cis_sqtl/about.

**KEY RESOURCES TABLE:** 

Resource Type	Specific Reagent or Resource	Source or Reference	Identifiers	Additional Information
Add additional rows as needed for each resource type	Include species and sex when applicable.	Include name of manufacturer, company, repository, individual, or research lab. Include PMID or DOI for references; use “this paper” if new.	Include catalog numbers, stock numbers, database IDs or accession numbers, and/or RRIDs. RRIDs are highly encouraged; search for RRIDs at https://scicrunch.org/resources.	Include any additional information or notes if necessary.
Deposited Data; Public Database	UK biobank Blood pQTLs	Published in https://www.nature.com/articles/s41586-023-06592-6	RRID:SCR_012815	
Deposited Data; Public Database	Wingo et. 2023 al Brain pQTLs	Published in https://www.nature.com/articles/s41591-023-02509-y#Sec10	NA	
Deposited Data; Public Database	Brain-Meta Brain methQTLs	Published in https://www.nature.com/articles/s41467-018-04558-1	NA	
Deposited Data; Public Database	GoDMC consortium Blood methQTLs	Published in https://www.nature.com/articles/s41588-021-00923-x	NA	
Deposited Data; Public Database	eQTLgen 1 Consortium Blood eQTLs	Published in https://www.nature.com/articles/s41588-021-00913-z	NA	
Deposited Data; Public Database	MetaBrain Consortium Brain eQTLs	Published in https://doi.org/10.1038/s41588-023-01300-6	NA	
Deposited Data; Public Database	Li et al., 2018 Blood sQTLs	Published in https://www.nature.com/articles/s41588-017-0004-9	NA	
Deposited Data; Public Database	Brain-Meta Brain sQTLs	https://www.nature.com/articles/s41588-022-01154-4	NA	
Deposited Data; Public Database	Kumasaka et al., 2019 Blood caQTLs	published in https://www.ncbi.nlm.nih.gov/pmc/articles/PMC6330062/	NA	
Deposited Data; Public Database	Zeng et al., 2024 Brain caQTLs	https://www.ncbi.nlm.nih.gov/pmc/articles/PMC10120699/	NA	
Deposited Data; Public Database	DrugBank data library (version 5.1.12)		RRID:SCR_002700	
Software; Algorithm	OPERA Software	Wu Y, Qi T, Wray NR, Visscher PM, Zeng J, Yang J. Joint analysis of GWAS and multi-omics QTL summary statistics reveals a large fraction of GWAS signals shared with molecular phenotypes. Cell Genomics. 2023;3(8):100344-.	NA	https://github.com/wuyangf7/OPERA/tree/main
