# Supplementary material for: Integrating Multi-Omics Summary Data Identifies Candidate Molecular Mechanisms for Major Depression
Source: Biol Psychiatry. Author manuscript; Available in PMC 2026 Aug 31. (PMC13527628; doi:10.1016/j.biopsych.2025.11.024)
Supplement: Supplementary file 1 [file NIHMS2187884-supplement-1.pdf]

SUPPLEMENTARY INFORMATION

Integrating Multiomic Summary Data Identifies Candidate Molecular Mechanisms for Major Depression

Nisbet *et al.*

Table of contents

1. List of Tables
2. Supplementary Methods
    a. Dataset information
    b. Functional Annotation analysis
    c. The OPERA tool
    d. Interpreting OPERA
    e. Comparing molecular phenotypes
3. Supplementary Results
    a. Significant Configurations
    b. Significant loci
    c. Proportion of significant results across molecular phenotypes
    d. Functional annotation
    e. Direction of effects
4. Supplementary Figures

List of Tables

(See separate Excel file)

Table S1. MD - Hypergeometric test inputs for druggable genome enrichment analysis

Table S2. Contingency tables for Fishers Exact test and Hypergeometric test inputs comparing MD, Schizophrenia and BPD

Table S3 Summary of schizophrenia and BPD result

Table S4. Genes overlapping between MD, SCZ and BPD in at least 1 omic type

Table S5. MD full OPERA output in blood

Table S6. MD full OPERA output in brain

Table S7. Schizophrenia full OPERA output in blood

Table S8. Schizophrenia full OPERA output in brain

Table S9. BPD full OPERA output in blood

Table S10. BPD full OPERA output in brain

Table S11. MD configurations – genes only - blood

Table S12. MD configurations – genes only – brain

Table S13. MD – all significant genes – blood and brain

Table S14. MD – Drug targets for significant MD genes in blood

Table S15. MD – Drug targets for significant MD genes in brain

Table S16. Schizophrenia – all significant genes – blood and brain

Table S17. BPD – all significant genes – blood and brain

Table S18. MD genes enriched in GWAS catalogue disorders

Table S19. SCZ genes enriched in GWAS catalogue disorders

Table S20. BPD genes enriched in GWAS catalogue disorders

Supplementary Methods

Dataset information

The MD GWAS was a meta-analysis and used phenotypes as defined from its component studies, excluding individuals with schizophrenia and BPD. In the whole sample the phenotype was defined in 4% by clinician interview, 54% from electronic health records, 14% questionnaire and 27% self-report (1).

Blood proteomic QTLs (pQTLs) were from the UK Biobank (n = 54,219) (2) with protein-plasma abundance measured using the antibody-based Olink Explore 3072 PEA. Summary statistics were available for 2,923 proteins, across which the study identified 14,287 SNP-protein associations (1,955 in cis, and 12,322 in trans). Information on the genes tagged by each probe were obtained from the UKB manifest accompanying the summary statistics.

Brain pQTLs were taken from Wingo et al. (3) using the combined male and female summary statistics (n = 1,277). The study meta-analysed protein abundance for 10,198 proteins in 5 post-mortem brain cohorts, all of which measured protein abundance using liquid chromatography coupled to tandem mass spectrometry (MS). We treated the tissue as 'bulk brain' as the published meta-analysis cohorts included samples from multiple brain regions. pQTLs were available for 8,044 proteins. The genes tagged by each protein measure was acquired using the Ensembl Biomart database ('hsapiens_gene_ensembl', hg19 version) (4).

Blood gene expression QTLs (eQTLs) were obtained from the eQTLgen consortium's meta-analysis of 19,250 gene expression profiles (n = 31,684)(5). The study incorporated 37 datasets (80.4% whole blood, 19.6% PBMCs), with gene expression quantified by Illumina and Affymetrix expression arrays as well as RNA sequencing. 88.3% of genes analysed had at least 1 cis-eQTL. Information on the genes tagged by each probe was acquired using the Ensembl Biomart database ('hsapiens_gene_ensembl', hg19 version) (4).

Brain eQTL summary statistics were obtained from the MetaBrain consortium's meta-analysis of post-mortem brain samples in 14 datasets (6) We used the QTLs derived from the cortex of European individuals (n = 2683) as this was the largest single sample available from MetaBrain. The study analysed 19,373 genes, of which 16,169 had at least one cis-eQTL in the cortex. Information on the genes tagged by each probe was also available from the summary statistic data.

The gene-splicing QTLs (sQTLs) for blood were derived from the GTEx sample for whole blood (n = 670) (7). The study used the LeafCutter tools to quantify the alternative splicing events for 9005 genes. 2735 genes have at least 1 cis -sQTL. Information on the genes tagged by each probe was also available from the summary statistic data.

The brain-derived sQTLs were obtained from the BrainMeta cohort, analysed by Qi et al. (8). The study analysed RNA-seq data using the THISTLE method, identifying 12,794 genes with at least one cis-sQTL. Information on the genes tagged by each probe was also available from the summary statistic data.

The DNA-methylation QTLs (methQTLs) in blood were obtained from the GoDMC consortium's meta-analysis of DNA methylation in whole blood or cord blood (n =32,851)(9). The study analysed 420,509 DNA methylation sites in 36 cohorts measured with Infinium HumanMethylation BeadChip arrays. Information on the genes tagged by each probe was

obtained from the InfiniumMethylation EPIC and 450k product Annotation files created by Illumina for the Infinium MethylationEPIC v1.0 and Infinium HumanMethylation450K v1.2 beadchips. The files list UCSC gene names which were treated as targets regions for assays, which we used to map specific CpGs to genes (10, 11).

The brain methQTLs were from the Brain-Meta cohort (n = 1160)(7). The study analysed 436,077 methylation sites in a meta-analysis of summary statistics from 5 cohorts of post-mortem brain samples. All methylation sites were measured using Illumina HumanMethylation450K array. Information on the genes tagged by each probe was obtained from the InfiniumMethylation BeadChips Annotation files (10, 11).

The chromatin-accessibility QTLs (caQTLs) in blood were derived from a study by Kumasaka et al. (n = 100) (12). The study analysed 277,128 chromatin accessibility peaks using ATAC-seq in lymphoblastoid cells. They identified over 15,000 SNP-peak associations. Probes were mapped to genes using the Ensembl Biomart tool . All genes within 10KB of the central position of a chromatin accessibility peak were mapped to each probe.

In brain, the caQTLs were obtained from a study conducted by Zeng et al (13). They analysed chromatin accessibility in postmortem brains (n = 616). Chromatin Accessibility was measured using ATAC-seq, identifying 34,539 open chromatin regions with caQTLs. Probes were mapped to genes using the Ensembl Biomart tool . All genes within 10KB of the central position of a chromatin accessibility peak were mapped to each probe.

Although all datasets were predominantly collected from independent studies. There was a degree of overlap between cohorts used to calculate xQTL datasets, in particular the ROSMAP and GTEx samples were used in multiple studies due to the limited availability of post-mortem brains. We used OPERA flags that account for correlations between datasets to account for overlap between QTL samples.

Harmonisation and processing pipelines will be available on the Psychiatric Genomics Consortium Functional Genomics group GitHub (https://github.com/psychiatric-genomics-consortium/FG_PipelineAndIntegration)

Functional Annotation analysis

We conducted functional annotation analyses of the significant genes from the OPERA analysis using the GENE2FUNC function in FUMA (14). We used the FUMA-implemented hypergeometric tests to test if the prioritised gene sets were overrepresented in gene sets defined from the GWAS catalogue, MsigDB c2 KEGG and Reactome databases, and the MsigDB c5 Gene Ontology databases (15). As with the drug targets we used background gene lists consisting of the genes mapped from all probes included in the analysis. Additionally we used the GENE2FUNC function to identify where our significant genes were overrepresented in mapped gene lists from the GWAS catalogue.

The OPERA tool

Box.1

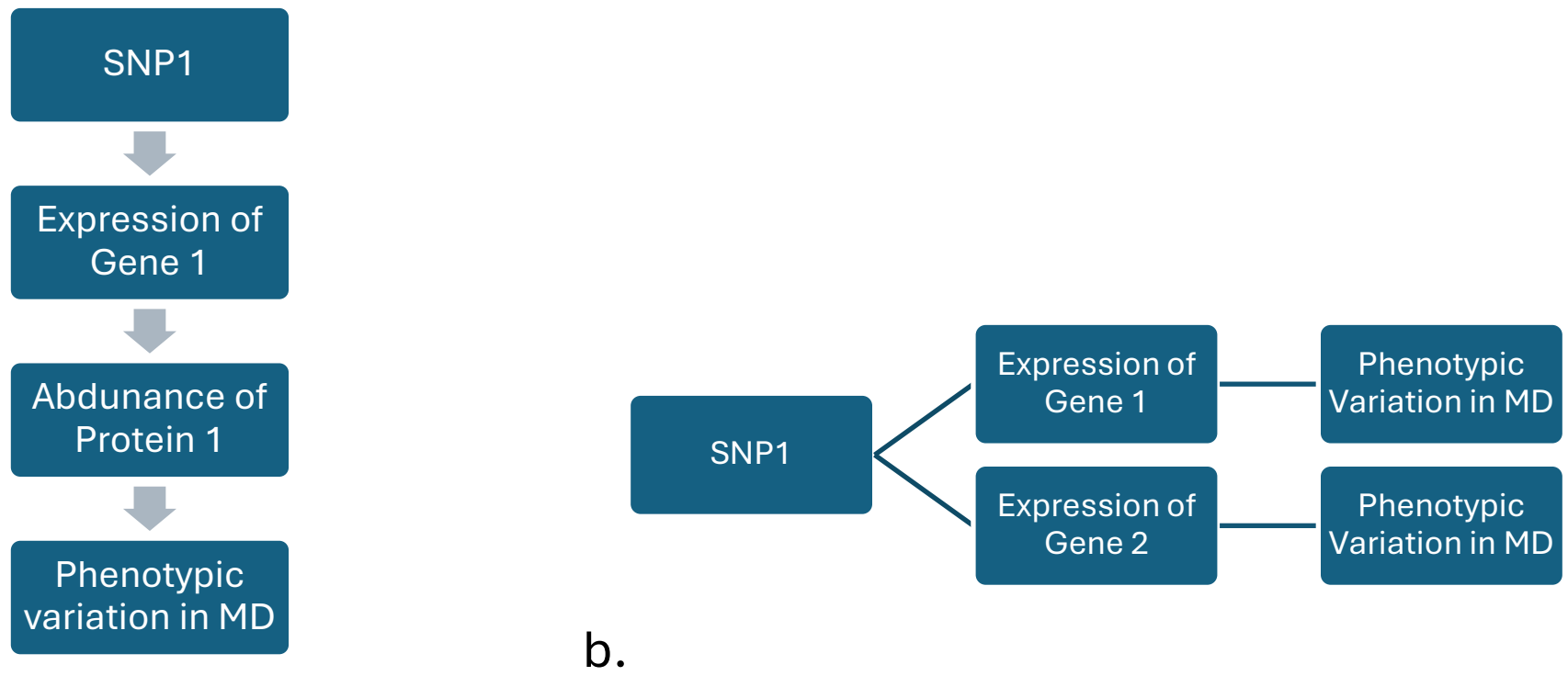

a. b.

Outline of vertical pleiotropy and horizontal pleiotropy

a) Depicts vertical pleiotropy, in which a genetic variant effects multiple phenotypes in a cascade of effects. b) Depicts horizontal pleiotropy in which genetic variants effect multiple traits directly.

In the OPERA analysis, multiple molecular phenotypes mapping to the same gene and which were significantly associated with MD using shared instruments were considered to be evidence of vertical pleiotropy.

For a full explanation of the statistical methodology of OPERA refer to Wu et al. (16). Briefly, OPERA expands on SMR to include multiple molecular phenotypes as exposures in a single analysis. It outputs configurations of omic-markers which, collectively, are significantly associated with a complex trait via a shared set of SNPs. The tool is designed to detect vertical pleiotropy by identifying groups of omic markers which map to a single gene and which are collectively associated with a complex trait using shared genetic instruments (see Box 1) .

To achieve this joint-SMR estimates for all probes are calculated. The likelihood of configurations of probes being collectively associated with the trait (referred to as the posterior probability of configuration/PPC) is then calculated based on the SMR estimates for each probe and the estimated prior probability of different configurations. Lastly, the posterior probability of association (PPA) for a configuration is then calculated from the sum of significant PPCs (see Box 2). Marginal PPAs are calculated from single-omic configurations, and joint PPAs for multi-omic configurations.

A multi-exposure HEIDI test is then run on results with a PPA > 0.9, this serves as a check that the instrument SNPs for each significant combination are shared across each probe and reject associations caused by linkage. This ensures that significant configurations are a result of pleiotropic-effects of a shared set of SNPs. Only configurations with a PPA > 0.9 and which pass the HEIDI test (i.e., p > 0.01) are considered to be significant evidence of a multi-omic causal association.

Box 2.

Equation 1.

$$PPC_y = \Pr(\delta = y | \hat{\boldsymbol{b}}, \boldsymbol{\pi}) = \frac{f(\hat{\boldsymbol{b}} | \delta = y) \times f(\delta = y | \pi_y)}{\sum_{k=1}^{\Omega} f(\hat{\boldsymbol{b}} | \delta = k) \times \pi_k}$$

Equation 2.

$$PPA_{joint} = \Pr(\boldsymbol{\lambda_c} = \mathbf{1} | \boldsymbol{b}, \hat{\pi})$$

Where δ represents a vector with values 1,2… Ω, indexing all configurations, with prior probabilities $\boldsymbol{\pi}$ (i.e. a vector of global probabilities for each configuration). $\hat{\boldsymbol{b}}$ represents a vector of SMR estimated effects of the exposures (i.e. each omic probe included in the configuration) on the outcome (MD). Applying these inputs to equation 1, the PPC can be interpreted as the probability of a specific set of beta values for probes in configuration y, multiplied by the prior probability of configuration y, divided by the sum of the probabilities for those sets of beta values for all possible configurations for these probes). This gives a posterior probability for a configuration of probes collectively associated with MD.

When effect sizes are extremely small PPC can be inflated, therefore a joint posterior probability of association (PPA) is calculated from the sum of significant PPCs. Equation 2 gives the formula where $\boldsymbol{\lambda_c}$ represents a dummy variable ($\in \{0,1\}$), indicating whether a specific exposure effects the outcome. The equation can be interpreted as the sum of all PPCs for which $\boldsymbol{\lambda_c}$ is equal to one, indicating the exposures significantly affect the trait of interest.

Interpreting the output

Simplified, example outputs can be seen in Table S1, alongside the outputs post gene-mapping. Both versions were used to determine the types of molecular phenotypes in each configuration as well as the genes to which the probes corresponded. Note in the example that for the locus on chromosome 11 all probes map to the same gene indicating likely vertical pleiotropy. However at the locus on chromosome 18, probes within a configuration map to multiple genes, meaning the evidence for vertical pleiotropy is weaker. This means that a 5-layer significant configuration was not necessarily evidence for a single gene to be associated with MD across 5 omic layers.

Additionally some genes (via their corresponding probes) showed associations with MD both across omics layers and across multiple probes within omics layers, for example multiple CpGs. The significant configurations in OPERA are limited to one probe per omic type. Therefore genes for which there were multiple significant probes of a single type, had multiple configurations. For example a gene may show association across a proteomic marker, a gene expression marker and three CpGs. In this scenario there would be 3 significant configurations

for that gene, each one sharing the same proteomic and expression marker, but with different CpGs (See Figure S11).

Although the raw output from the analysis refers to molecular phenotypes, we chose to focus the majority of the interpretation on the gene mapped from each probe in order to better identify where cascading molecular associations (i.e. vertical pleiotropy) were present.

[FIGURE S11]

Comparing molecular phenotypes

Our power to detect significant associations between omic markers and MD varied between omic types, tissues and disorders, limiting our ability to compare them directly. The main contributors to this included:

Sample overlap – In blood, xQTL samples were largely derived from independent samples. There was, however, considerable overlap in the datasets provided in post-mortem brain, due to their greater scarcity. It is possible that the lack of overlap in the blood reduces the likelihood of identifying multi-omic signals due to between-sample variation. However, it may also reduce the likelihood of sample specific confounding factors. Although there are challenges around the development and harmonisation of high-quality and well-powered QTL data across multiple molecular layers, our study highlights that these developing areas are much needed, if we are to advance our understanding of molecular functions of GWAS findings.

Probe coverage – Not all genes included in the analysis were mapped to all five omic types. In particular, the 400,000+ methylation probes mapped many more genes than the fewer than 3000 proteins measured in the blood, especially in gene dense regions. This results in an increased number of genes with single-omic associations, especially for DNA methylation (see Figure 1).

xQTL sample size – Power to accurately detect associations between SNPs and molecular phenotypes is dependent on sample size. As an extension of this the ability for SMR (on which OPERA is based) to accurately test for associations between molecular phenotypes and a complex trait are dependent on the power of the QTL and GWAS summary statistics. Variation in sample sizes across tissues, and molecular phenotypes mean that our power to detect associations varied also. This confounds formal tests that compare the relative importance of different molecular phenotypes in depression.

Number of probes – DNA methylation summary statistics used in our analysis had a greater number of CpGs per locus relative to other molecular phenotypes. This was also true to a lesser extent of splicing variation. Although OPERA accounts for the relative proportions of each molecular phenotype in the analysis, high linkage disequilibrium means there was a higher likelihood of causal SNPs effecting multiple CpGs, leading to an inflation in the number of DNA methylation measurements associated with depression relative to other phenotypes. Similarly, splicing and DNA Methylation probes were able to map multiple probes to the same gene. This results in a higher number of probe-associations relative to other molecular phenotypes (assuming sufficient power), and a higher likelihood of single-omic associations.

Gene mapping – measures of protein abundance, splicing variants, and gene expression can all be mapped directly to individual genes with high confidence, however DNA methylation and chromatin accessibility are mapped using statistical methods such as fine mapping, and nearest gene. When interpreting the results in terms of genes associated with MD, genes associated primarily via DNA methylation or chromatin accessibility markers are less reliably associated relative to other molecular phenotypes.

Supplementary results

Significant configurations summary

In the brain, the 1405 significant probes were associated with MD in 3737 configurations (PPA > 0.90 and pHEIDI > 0.01). 36 significant configurations of probes included convergent evidence from 4 different molecular layers/types, 342 included 3 types, 1468 included two types and 1891 included 1 type. Note that overlapping loci, meant in some cases single

In the blood-based analysis the 1877 probes were significantly associated with MD in 4953 configurations (PPA > 0.90 and pHEIDI > 0.01). 1 configuration included all 5 omic types, 58 included 4 omic types, 821 included 3 types, 1600 included two types and 2473 included 1 type.

Significant loci

In the brain-derived xQTLs, significant omic or multi-omic associations were found across 295 risk loci (45.4% of the genome wide significant loci for MD). In the blood xQTLs, we found associations across 317 risk loci (48.9% of the genome wide significant loci for MD). 205 loci had significant associations in both analyses (69.4%, of the brain loci, 64.6% of the blood loci).

Proportion of significant results across molecular phenotypes

We compared the proportions of significant probes from each dataset to investigate the relative contribution of molecular phenotypes in our results. We considered all probes included in a significant configuration as being significantly associated with MD (PPA > 0.90 and pHEIDI > 0.01).

From the blood-derived datasets we observed significant associations for 13 proteomic probes (0.46%), 127 gene expression probes (0.69%), 45 gene splicing probes (0.07%), 1608 methylation sites (0.77%), and 84 chromatin accessibility peaks (0.03%, Figure 1).

From the brain-derived datasets we observed significant associations for 29 proteomic probes (0.36%), 109 gene expression probes (0.59%), 470 gene splicing probes (0.76%), 750 methylation sites (0.17%), and 47 chromatin accessibility peaks (0.02%) (Figure S1).

Functional Annotation

We conducted functional annotation using the GENE2FUNC function in FUMA on the gene lists obtained from mapping OPERA input probes.

In FUMA's gene set enrichment analysis we found our prioritised gene lists to be enriched in several gene sets (Figures S12-15).

The blood-derived priority genes were enriched in 12 GO biological process gene sets. The most significant pathways were Neuron development ($p = 1.26 \times 10^{-6}$), Homophilic adhesion via plasma membrane ($p = 3.23 \times 10^{-6}$) and synapse organisation ($p = 3.78 \times 10^{-6}$). Additionally, they were enriched in 21 GO cellular component gene-sets the most significant being Postsynaptic specialisation ($p = 1.31 \times 10^{-6}$)

The brain-derived priority genes were significantly enriched in 30 Gene Ontology (GO) biological process gene sets from the Molecular Signatures Database/MSigDB c5 (15), the top 3 being: Cell adhesion via plasma membrane adhesion molecules ($p = 1.13 \times 10^{-10}$), Homophilic adhesion via plasma membrane ($p = 1.30 \times 10^{-9}$) and Synapse Assembly ($p = 2.69 \times 10^{-9}$). Additionally, they were enriched in 25 GO cellular component gene-sets the top one being Dendritic tree ($p = 1.62 \times 10^{-8}$)

In addition our significant gene lists were enriched in GWASs implicating genes across a range of disorders. The full results are reported in Tables S18-20.

Direction of effects

For the genes significantly associated with MD, via both gene expression and protein abundance we compared the direction of effects. With the exception of *DUSP13B*, SMR effect sizes for pQTL and eQTL were highly correlated ($r = 0.97$). For *DUSP13B* gene expression showed a positive association with MD and protein abundance showed a small negative association. The reason for this is unclear, however a possible explanation is that neither eQTLGen nor UKB specify whether the measured protein or transcript corresponded to *DUSP13A* or *DUSP13B*, the two main protein products of the *DUSP13* gene; *DUSP13A* and *DUSP13B* share only 42% of amino acid sequences, and differing measuring technologies between the cohorts could be capturing different proportions of each (17).

Supplementary Figures

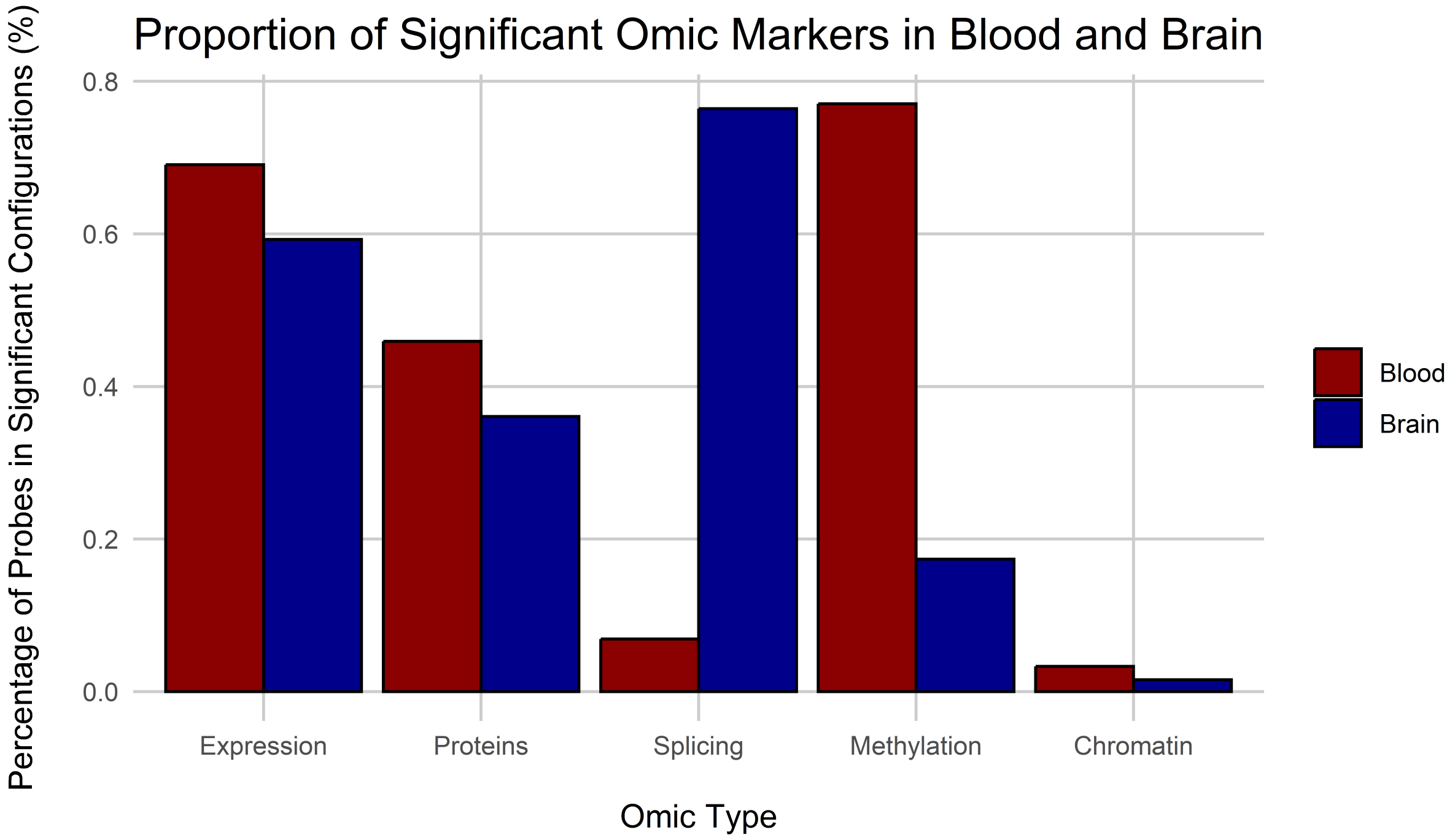

Figure S1. Plot depicting the percentage of probes for which there is a significant association of that probe type with MD. Note that this is biased by the power of the xQTL sample to detect effects.

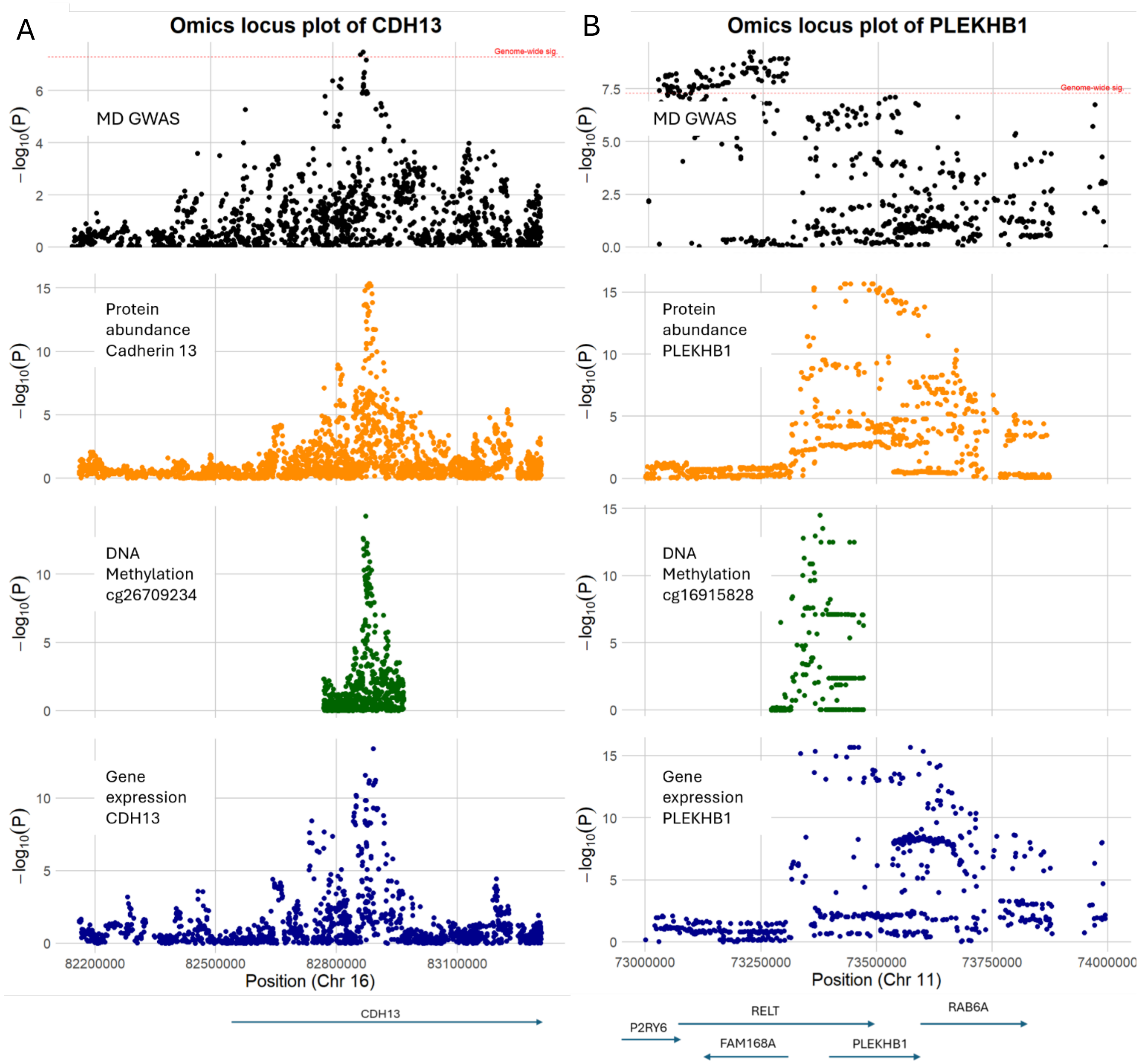

Figure S2. Example multi-omic SMR plots demonstrating xQTL associations at multiple molecular phenotypes in A) CDH13 and B) PLEKHB1 in the brain. CDH13 demonstrates clear association patterns consistent with vertical pleiotropy in which the SNPs contributing to MD pathophysiology are likely doing so via multiple molecular phenotypes. However PLEKHB1 is in a gene dense region and so the association peaks could be influenced by multiple genes. Note - these genes were selected from our high confidence-genes to demonstrate the differing association patterns in gene dense regions

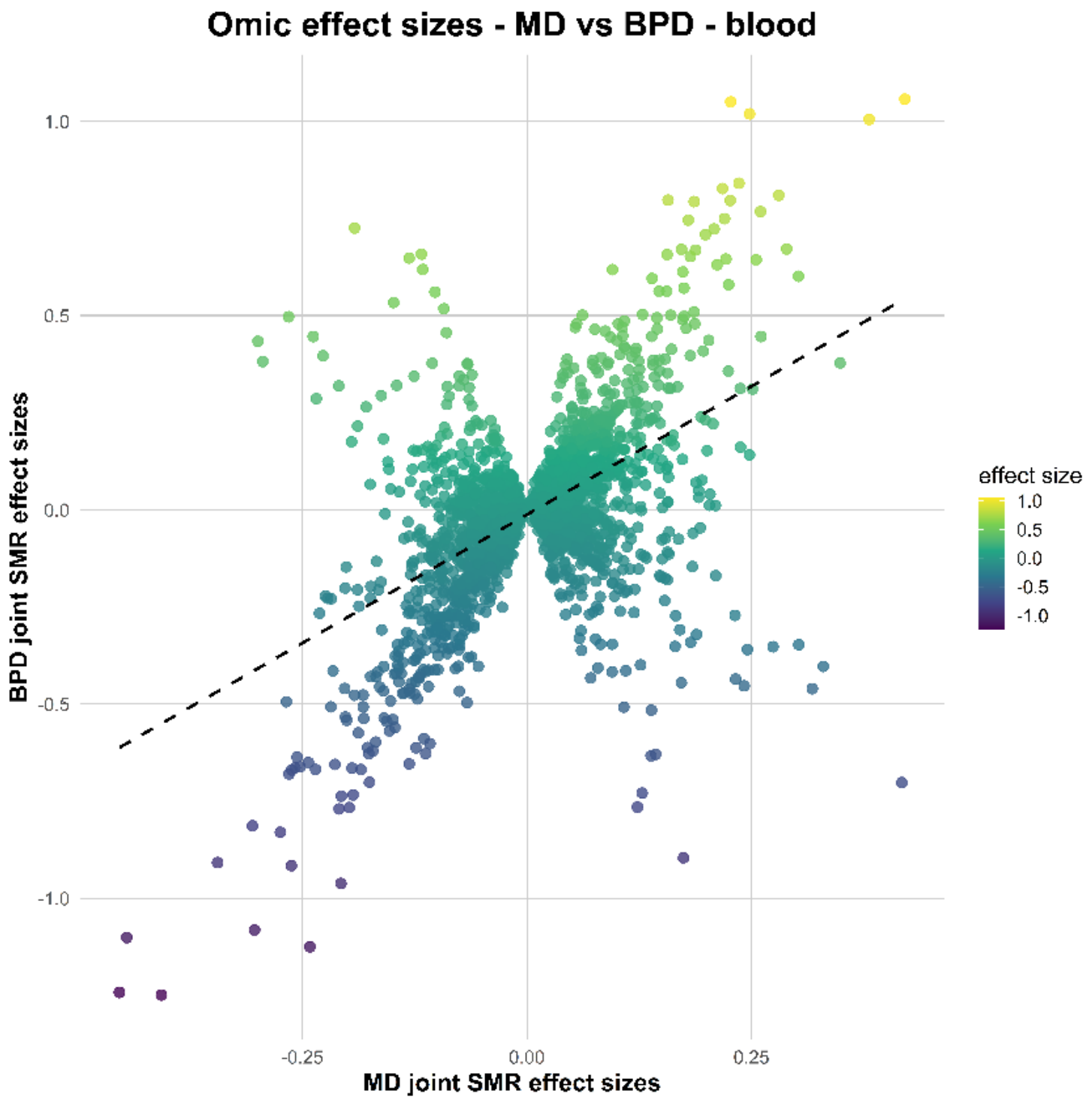

Figure S3 SMR effect sizes – MD against BPD in blood (MD is filtered to results with $p < 0.05$)

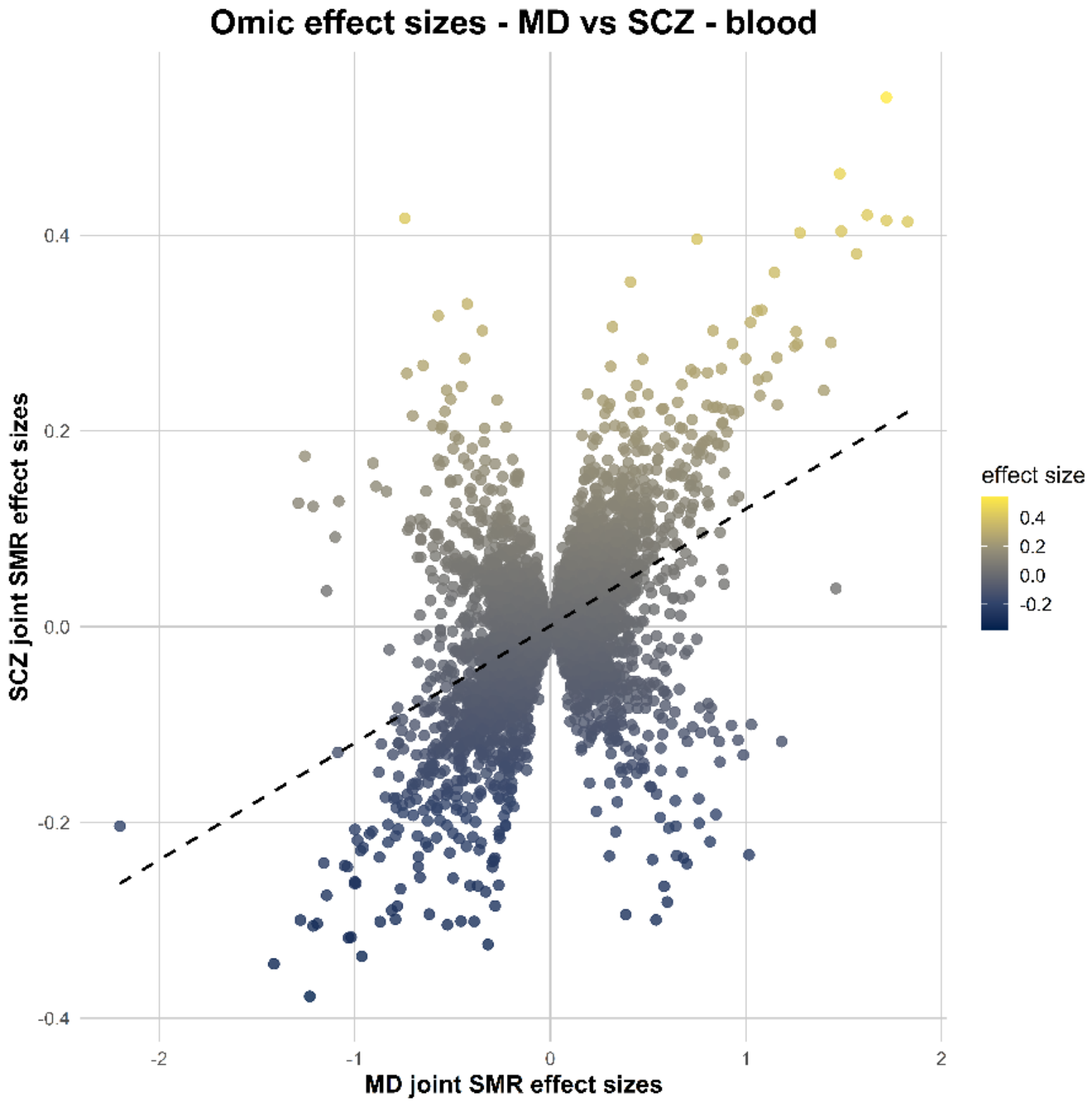

Figure S4 SMR effect sizes – MD against SCZ in blood (MD is filtered to results with $p < 0.05$)

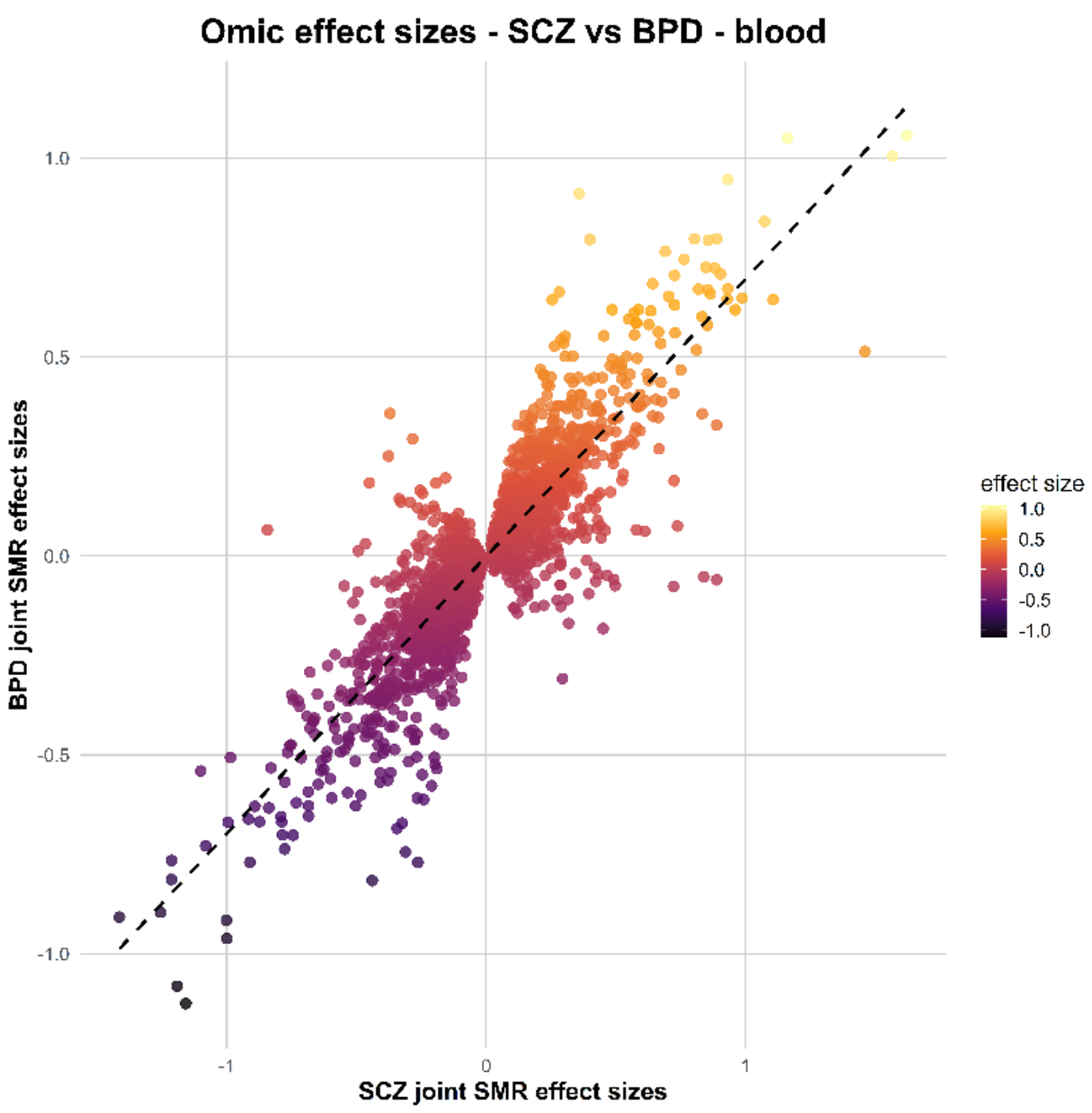

Figure S5 SMR effect sizes – SCZ against BPD in blood (SCZ is filtered to results with $p < 0.05$)

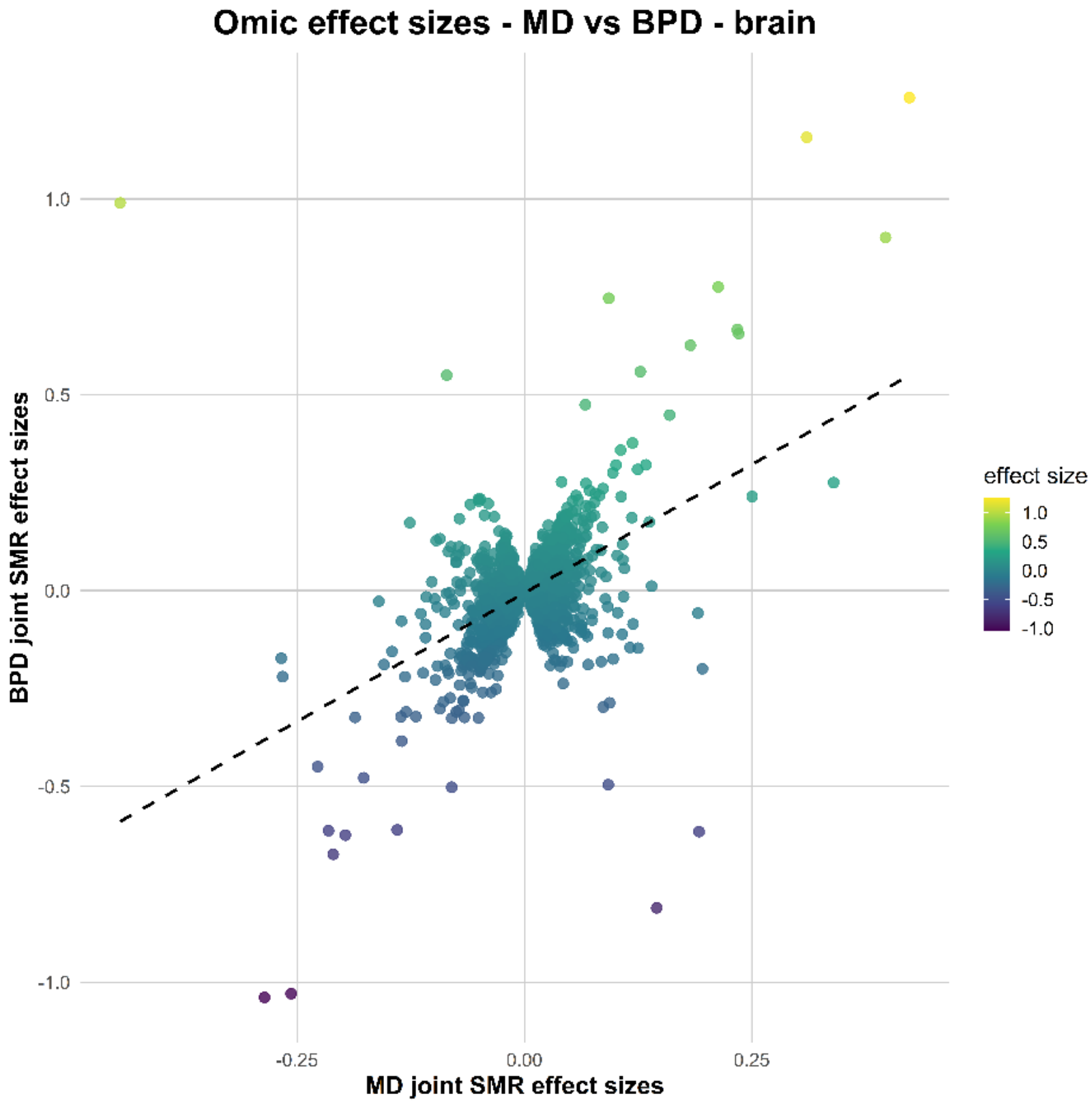

Figure S6 SMR effect sizes – MD against BPD in brain (MD is filtered to results with p < 0.05)

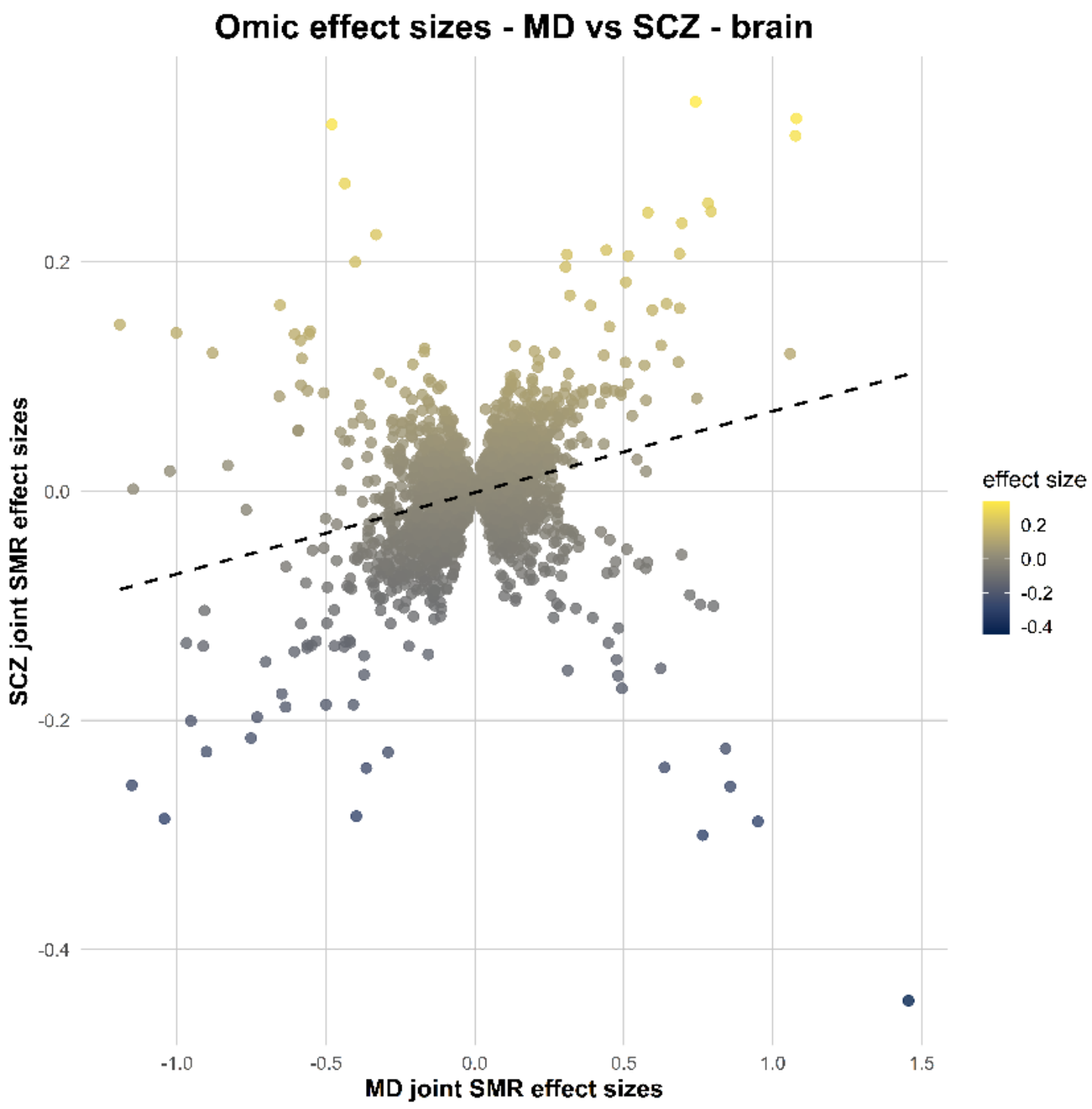

Figure S7 SMR effect sizes – MD against SCZ in brain (MD is filtered to results with p < 0.05)

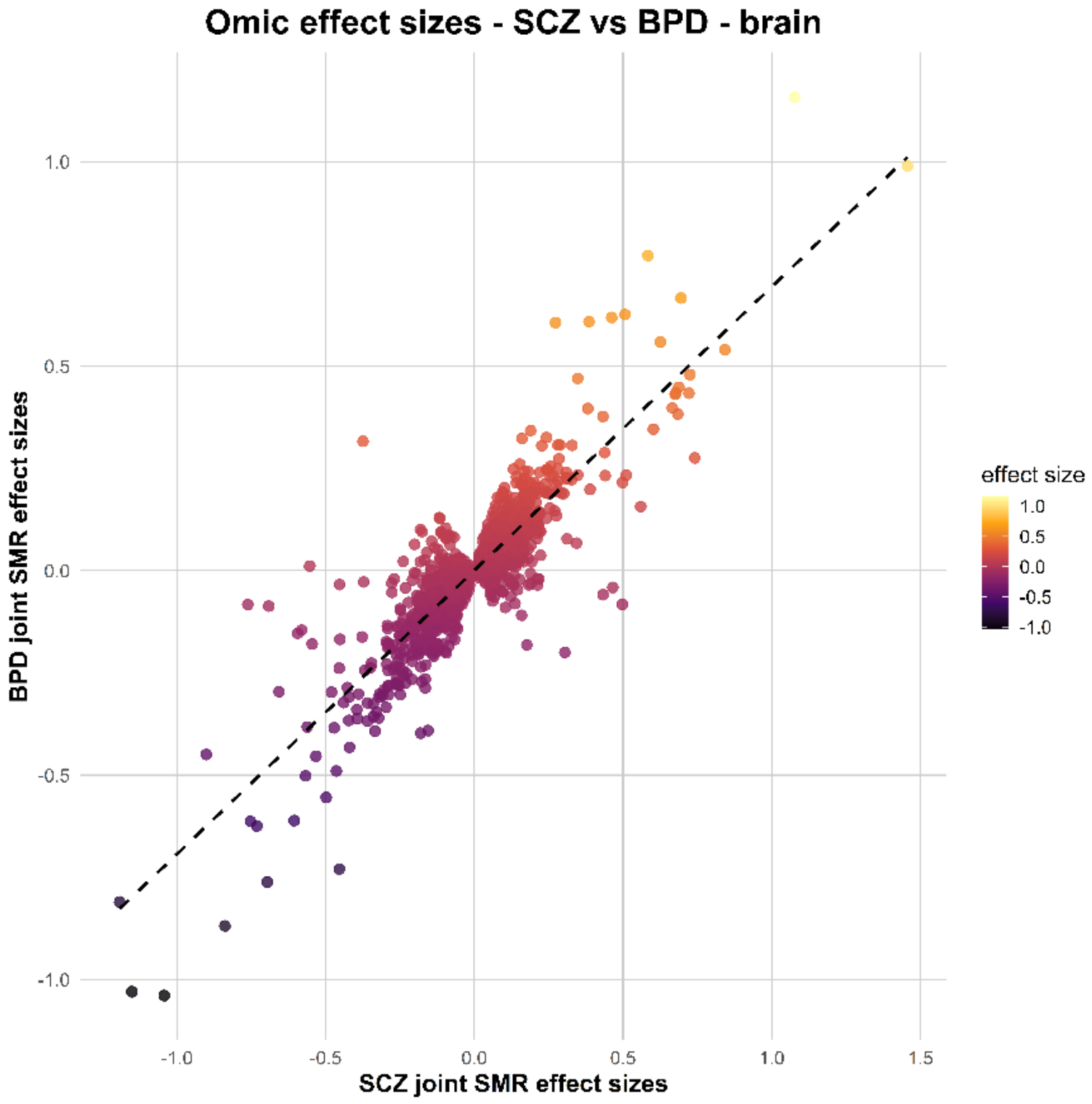

Figure S8 SMR effect sizes – SCZ against BPD in brain (SCZ is filtered to results with p < 0.05)

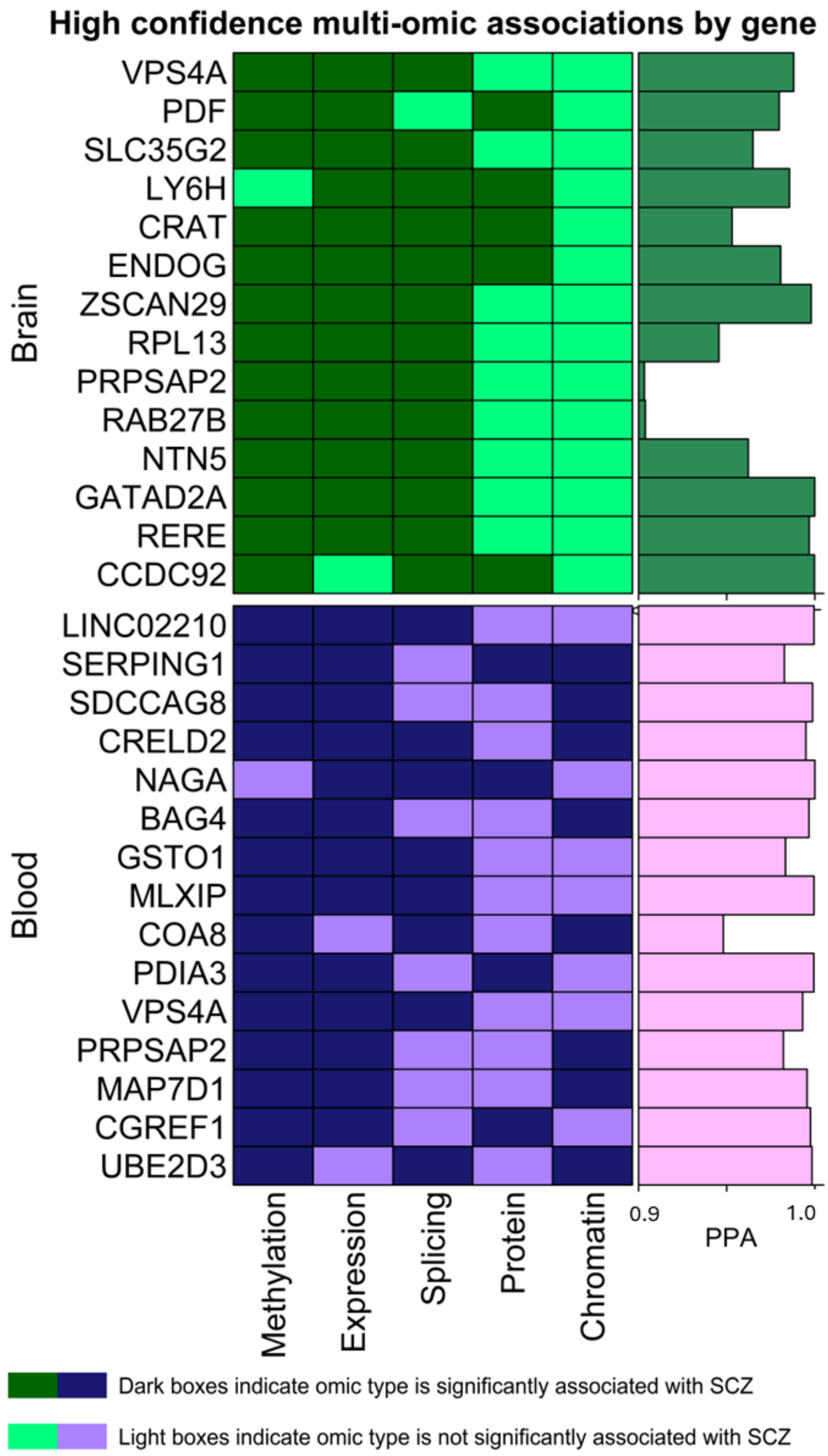

Figure S9 High confidence configurations for schizophrenia.

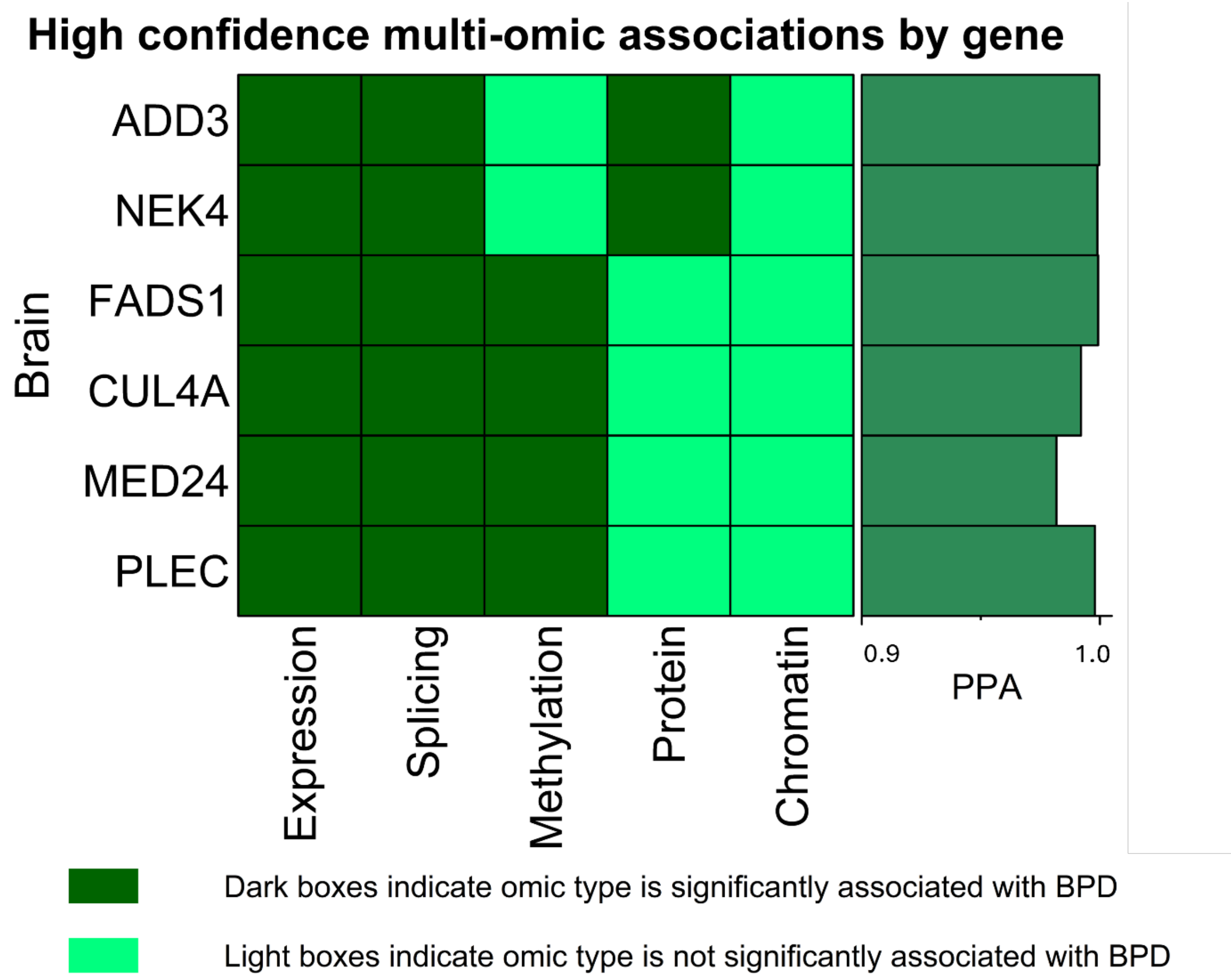

Figure S10 *High confidence configurations for BPD. Note no high confidence genes were observed in the blood*

Chr	GWAS SNP	Expo1_ID	Expo2_ID	Expo3_ID	Expo4_ID	PPA(1,2,3,4)	pHEIDI(1,2,3,4)
11	rs7114376	ENSG00000021300.14_brain_eqtl	cg16915828_brain_methqtl	chr11:73371897:73372019:clu_291882_brain_sqtl	Q9UF11_brain_pqtl	0.995936	6.85E-02
18	rs1623060	ENSG00000141452.10_brain_eqtl	cg14672496_brain_methqtl	chr18:21106729:21109143:clu_23929_brain_sqtl	Q96DM3_brain_pqtl	0.999912	1.53E-01

Chr	GWAS SNP	Expo1_ID	Expo2_ID	Expo3_ID	Expo4_ID	PPA(1,2,3,4)	pHEIDI(1,2,3,4)
11	rs7114376	PLEKHB1	PLEKHB1	PLEKHB1	PLEKHB1	0.995936	0.068475
18	rs1623060	RMC1	NPC1	RMC1	RMC1	0.999912	1.53E-01

Figure S11 – Example output of OPERA and the corresponding genes to which each probe maps. Expo-columns refer to different omics markers, PPA = posterior probability of colocalization across all omic layers, pHEIDI = p value for HEIDI test.

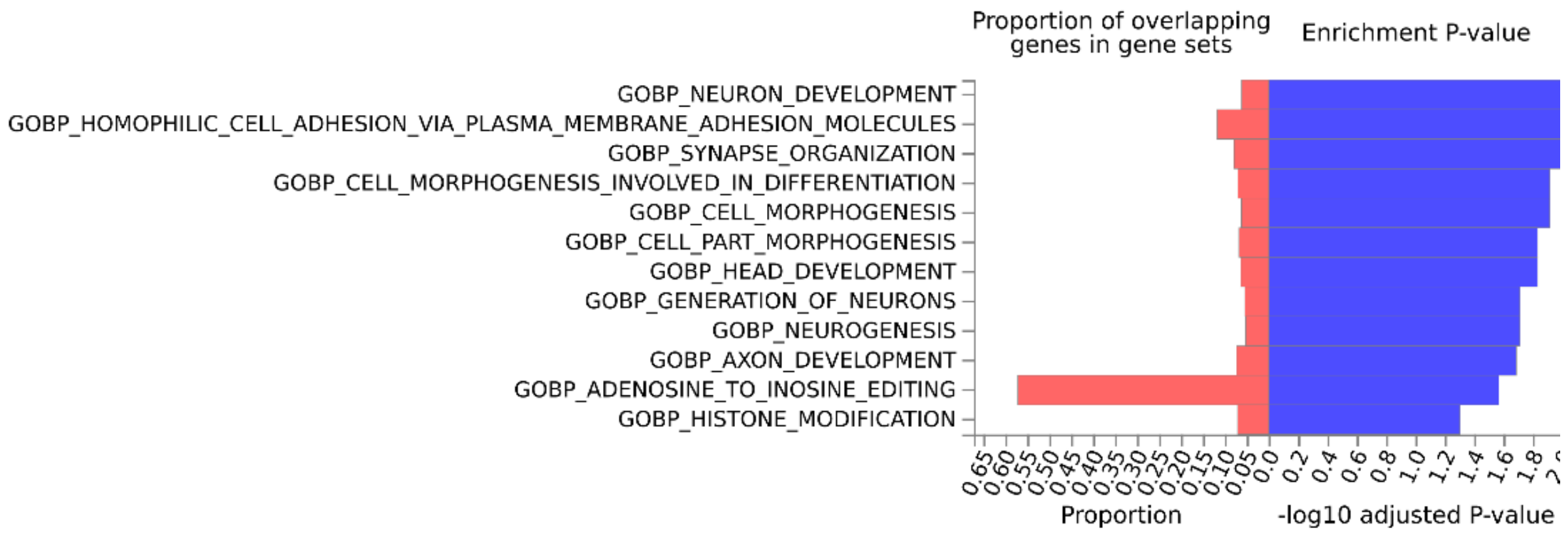

Figure S12. Gene Ontology biological processes in blood

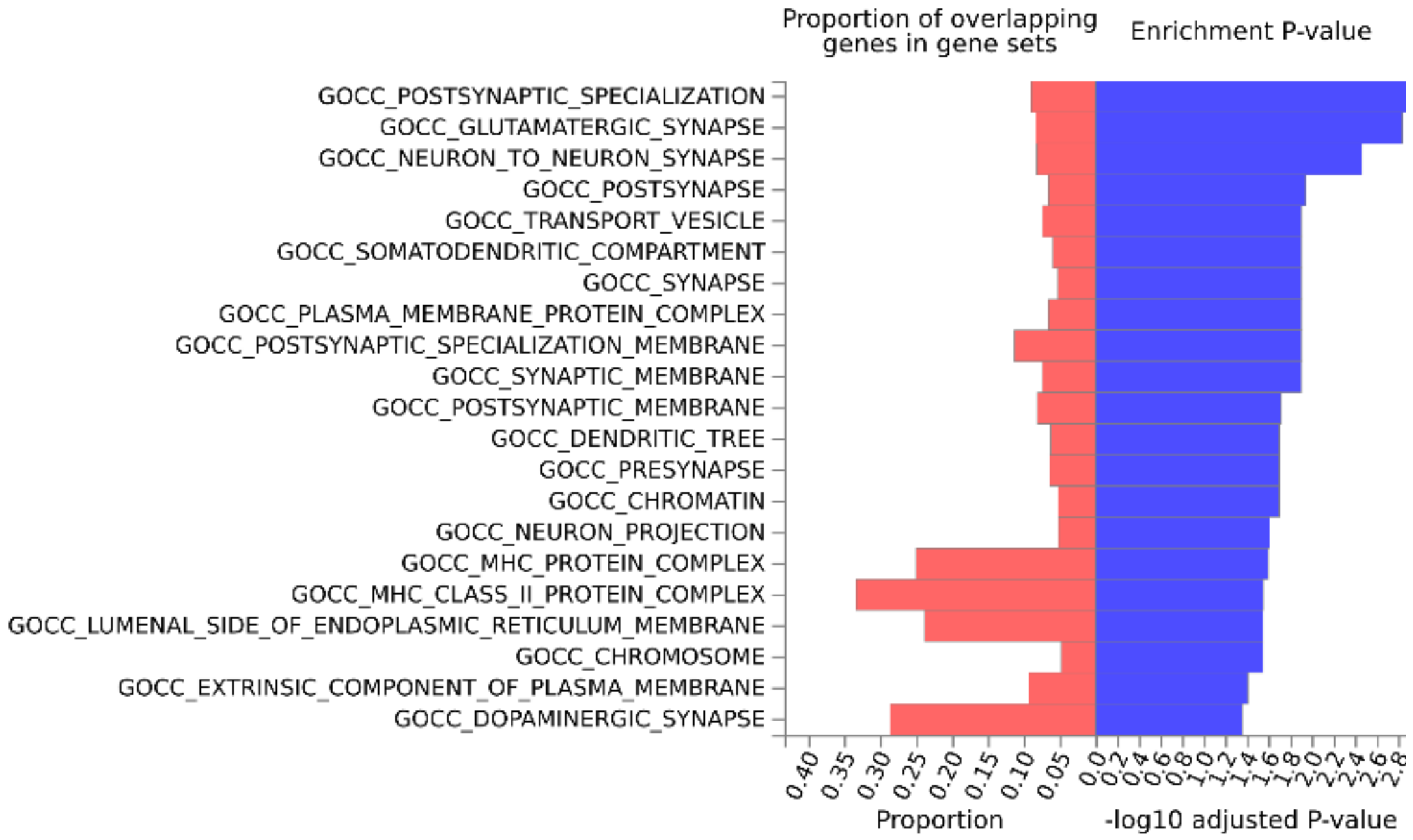

Figure S13. Gene Ontology cellular components blood

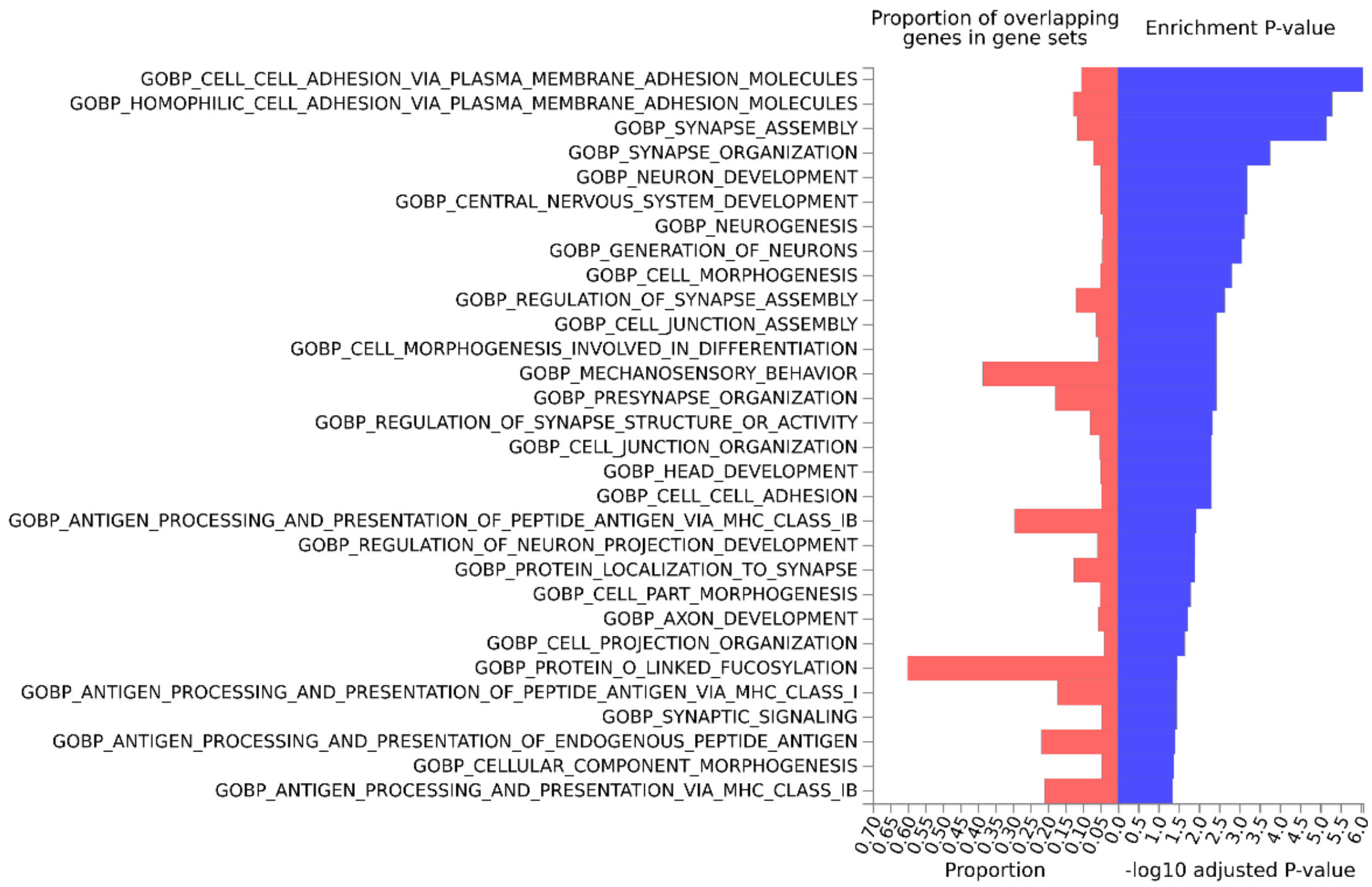

Figure S14. Gene Ontology biological processes brain

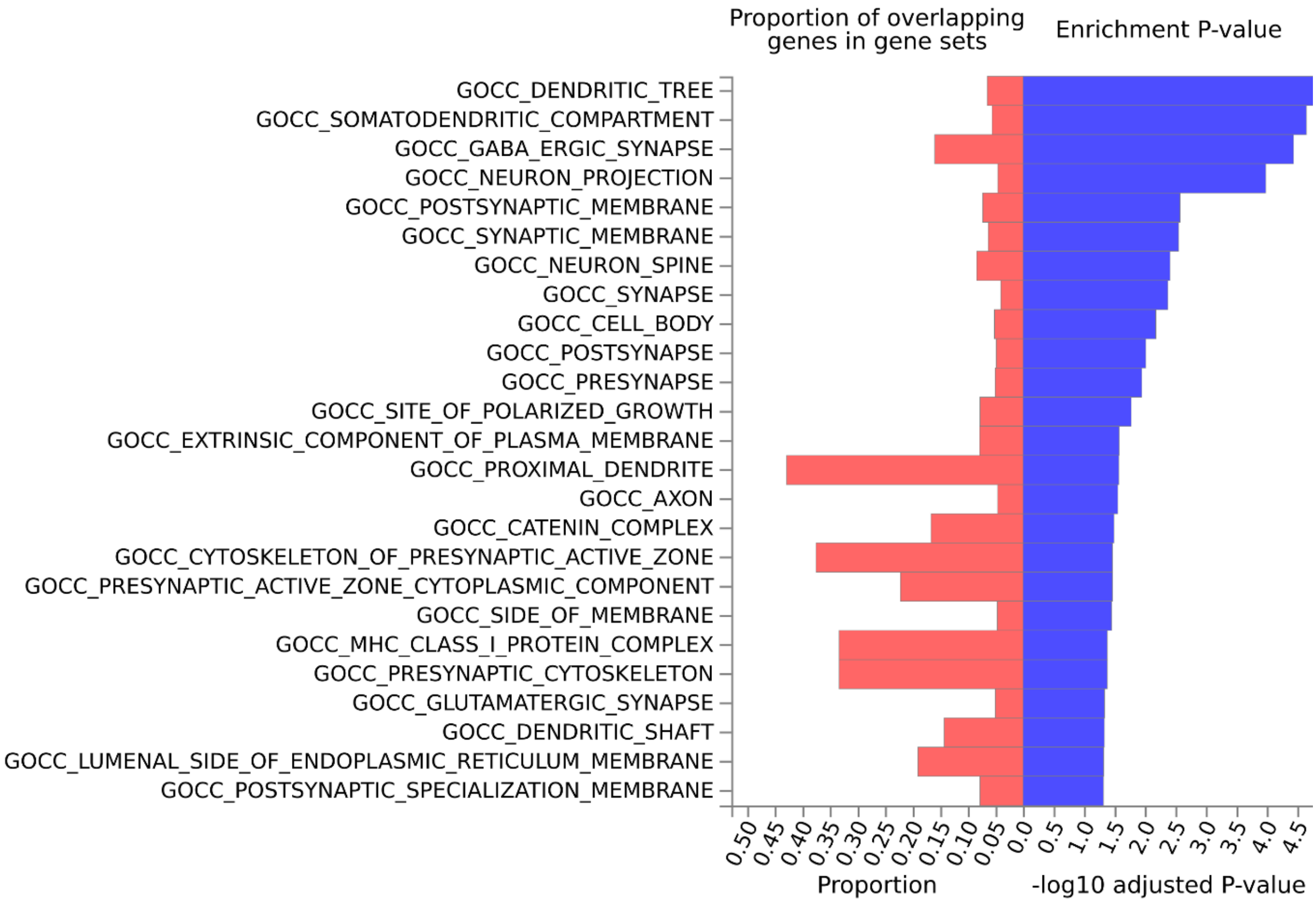

Figure S15 Gene Ontology cellular components brain

References

1. Major Depressive Disorder Working Group of the Psychiatric Genomics Consortium. Electronic address ameau, Major Depressive Disorder Working Group of the Psychiatric Genomics C. Trans-ancestry genome-wide study of depression identifies 697 associations implicating cell types and pharmacotherapies. Cell. 2025;188(3):640-52 e9.
2. Sun BB, Chiou J, Traylor M, Benner C, Hsu Y-H, Richardson TG, et al. Plasma proteomic associations with genetics and health in the UK Biobank. Nature. 2023;622(7982):329-38.
3. Wingo AP, Liu Y, Gerasimov ES, Vattathil SM, Liu J, Cutler DJ, et al. Sex differences in brain protein expression and disease. Nature Medicine. 2023;29(9):2224-32.
4. Smedley D, Haider S, Ballester B, Holland R, London D, Thorisson G, et al. BioMart--biological queries made easy. BMC Genomics. 2009;10:22.
5. Võsa U, Claringbould A, Westra H-J, Bonder MJ, Deelen P, Zeng B, et al. Large-scale cis- and trans-eQTL analyses identify thousands of genetic loci and polygenic scores that regulate blood gene expression. Nature Genetics. 2021;53(9):1300-10.
6. de Klein N, Tsai EA, Vochteloo M, Baird D, Huang Y, Chen C-Y, et al. Brain expression quantitative trait locus and network analyses reveal downstream effects and putative drivers for brain-related diseases. Nature Genetics. 2023;55(3):377-88.
7. Qi T, Wu Y, Zeng J, Zhang F, Xue A, Jiang L, et al. Identifying gene targets for brain-related traits using transcriptomic and methylomic data from blood. Nature Communications. 2018;9(1):2282-.
8. Qi T, Wu Y, Fang H, Zhang F, Liu S, Zeng J, et al. Genetic control of RNA splicing and its distinct role in complex trait variation. Nature Genetics. 2022;54(9):1355-63.
9. Min JL, Hemani G, Hannon E, Dekkers KF, Castillo-Fernandez J, Luijk R, et al. Genomic and phenotypic insights from an atlas of genetic effects on DNA methylation. Nature Genetics. 2021;53(9):1311-21.
10. Illumina. Infinium MethylationEPIC Manifest Column Headings: Illumina, Inc.; 2013 [Available from: https://knowledge.illumina.com/microarray/general/microarray-general-reference_material-list/000001568.
11. Illumina. Infinium HumanMethylation450K BeadChip Product Files 2013 [Available from: https://emea.support.illumina.com/array/array_kits/infinium_humanmethylation450_beadchip_kit/downloads.html.
12. Kumasaka N, Knights AJ, Gaffney DJ. High-resolution genetic mapping of putative causal interactions between regions of open chromatin. Nature Genetics. 2019;51(1):128-37.
13. Zeng B, Bendl J, Deng C, Lee D, Misir R, Reach SM, et al. Genetic regulation of cell type–specific chromatin accessibility shapes brain disease etiology. Science. 2024;384(6698):eadh4265.
14. Watanabe K, Taskesen E, van Bochoven A, Posthuma D. Functional mapping and annotation of genetic associations with FUMA. Nat Commun. 2017;8(1):1826.
15. Liberzon A, Subramanian A, Pinchback R, Thorvaldsdóttir H, Tamayo P, Mesirov JP. Molecular signatures database (MSigDB) 3.0. Bioinformatics. 2011;27(12):1739-40.
16. Wu Y, Zeng J, Zhang F, Zhu Z, Qi T, Zheng Z, et al. Integrative analysis of omics summary data reveals putative mechanisms underlying complex traits. Nature Communications. 2018;9(1):918-.
17. Park JE, Park BC, Kim HA, Song M, Park SG, Lee DH, et al. Positive regulation of apoptosis signal-regulating kinase 1 by dual-specificity phosphatase 13A. Cell Mol Life Sci. 2010;67(15):2619-29.
